# Anaerobic Degradation of Non-Methane Alkanes by “*Candidatus* Methanoliparia” in Hydrocarbon Seeps of the Gulf of Mexico

**DOI:** 10.1128/mBio.01814-19

**Published:** 2019-08-20

**Authors:** Rafael Laso-Pérez, Cedric Hahn, Daan M. van Vliet, Halina E. Tegetmeyer, Florence Schubotz, Nadine T. Smit, Thomas Pape, Heiko Sahling, Gerhard Bohrmann, Antje Boetius, Katrin Knittel, Gunter Wegener

**Affiliations:** a Max-Planck Institute for Marine Microbiology, Bremen, Germany; b Alfred Wegener Institute Helmholtz Center for Polar and Marine Research, Bremerhaven, Germany; c MARUM, Center for Marine Environmental Sciences and Department of Geosciences, University of Bremen, Bremen, Germany; d Laboratory of Microbiology, Wageningen University and Research, Wageningen, The Netherlands; e Center for Biotechnology, Bielefeld University, Bielefeld, Germany; Bigelow Laboratory; California Institute of Technology

**Keywords:** alkane degradation, archaea, methanogenesis, methyl-coenzyme M reductase, oil seeps

## Abstract

Crude oil and gases in the seabed provide an important energy source for subsurface microorganisms. We investigated the role of archaea in the anaerobic degradation of non-methane alkanes in deep-sea oil seeps from the Gulf of Mexico. We identified microscopically the ethane and short-chain alkane oxidizers “*Candidatus* Argoarchaeum” and “*Candidatus* Syntrophoarchaeum” forming consortia with bacteria. Moreover, we found that the sediments contain large numbers of cells from the archaeal clade “*Candidatus* Methanoliparia,” which was previously proposed to perform methanogenic alkane degradation. “*Ca.* Methanoliparia” occurred abundantly as single cells attached to oil droplets in sediments without apparent bacterial or archaeal partners. Metagenome-assembled genomes of “*Ca.* Methanoliparia” encode a complete methanogenesis pathway including a canonical methyl-coenzyme M reductase (MCR) but also a highly divergent MCR related to those of alkane-degrading archaea and pathways for the oxidation of long-chain alkyl units. Its metabolic genomic potential and its global detection in hydrocarbon reservoirs suggest that “*Ca.* Methanoliparia” is an important methanogenic alkane degrader in subsurface environments, producing methane by alkane disproportionation as a single organism.

## Abstract (precis)

Oil-rich sediments from the Gulf of Mexico were found to contain diverse alkane-degrading groups of archaea. The symbiotic, consortium-forming “*Candidatus* Argoarchaeum” and “*Candidatus* Syntrophoarchaeum” are likely responsible for the degradation of ethane and short-chain alkanes, with the help of sulfate-reducing bacteria. “*Ca.* Methanoliparia” occurs as single cells associated with oil droplets. These archaea encode two phylogenetically different methyl-coenzyme M reductases that may allow this organism to thrive as a methanogen on a substrate of long-chain alkanes. Based on a library survey, we show that “*Ca. Methanoliparia*” is frequently detected in oil reservoirs and may be a key agent in the transformation of long-chain alkanes to methane. Our findings provide evidence for the important and diverse roles of archaea in alkane-rich marine habitats and support the notion of a significant functional versatility of the methyl coenzyme M reductase.

## INTRODUCTION

Archaea are key players in the global carbon, nitrogen, and sulfur cycle (1). In particular, methanogenic archaea have been important for the Earth’s climate through time, as their metabolic product, methane, is an important greenhouse gas that is 25 times more potent than CO_2_ (2). All cultured methanogens belong to the *Euryarchaeota* phylum and produce methane either from the reduction of carbon dioxide with hydrogen or formate, the disproportionation of acetate, or the use of methylated substrates. The key enzyme of this process is methyl-coenzyme M (CoM) reductase (MCR), which catalyzes the final step in methane formation (3). Marine sediments contain large amounts of methane, formed primarily by microbial methanogenesis (4). The thermocatalytic degradation of organic matter also produces methane and other short-chain alkanes, such as ethane, propane, and butane, as well as liquid compounds (crude oil) (5). It has been speculated that short-chain alkanes like ethane and propane can also be produced microbiologically, but the potential microorganisms have not been obtained in culture for assessing the respective biochemical pathways (6).

Hydrocarbons tend to migrate from deep organic-substance-rich source rocks toward the sediment surface. However, a large fraction, primarily alkanes, is already degraded within anoxic sediment layers (7, 8). The anaerobic oxidation of methane (AOM) is performed by different clades of anaerobic methanotrophic archaea (ANME), which are close relatives of the methanogens (9). ANME use the methanogenesis pathway in a reverse direction to oxidize methane. AOM is coupled mostly with sulfate reduction performed by bacterial partners (10–15). However, some ANME may thrive without partners in the presence of other electron acceptors, such as iron, manganese, and nitrate (16–20).

The anaerobic degradation of other alkanes was for a long time exclusively assigned to bacteria. Most of these bacteria are affiliated with the Deltaproteobacteria class, and they use the addition to fumarate predominantly to activate alkane molecules (21–23). Some of these bacteria, like specific strains of *Smithella* spp. and *Syntrophus* spp. (both members of the *Syntrophobacterales*), degrade alkanes in consortia with methanogens (24, 25).

Recently, several novel archaea have been shown to degrade non-methane alkanes anaerobically using mechanisms of archaeal origin, proving that alkane activation is not limited to bacteria. First, thermophilic archaea of the “*Candidatus* Syntrophoarchaeum” clade were shown to degrade short-chain alkanes. They use divergent MCRs to activate propane and *n-*butane with CoM, forming CoM-bound alkyl units as primary intermediates (26). In a so far unresolved step, “*Ca.* Syntrophoarchaeum” archaea oxidize the alkyl groups to coenzyme A (CoA)-bound fatty acids, which are then completely oxidized to CO_2_. The alkane oxidation in “*Ca.* Syntrophoarchaeum” is coupled to sulfate reduction performed by the bacterial partner “*Candidatus* Desulfofervidus auxilii,” as shown for the thermophilic AOM (27). Recently, anaerobic ethane oxidation by the psychrophilic archaeon “*Candidatus* Argoarchaeum ethanivorans” (affiliated with the GoM-Arc1 clade, where “GoM” stands for “Gulf of Mexico”) was described (28). Similarly to “*Ca.* Syntrophoarchaeum,” “*Ca.* Argoarchaeum ethanivorans” activates ethane using divergent MCRs forming CoM-bound ethyl units that are, after transformation to acetyl units, completely oxidized in the Wood-Ljungdahl pathway. “*Ca.* Argoarchaeum ethanivorans” is also associated with sulfate-reducing partner bacteria (28).

Additionally, several metagenomic studies revealed the presence of highly modified MCRs in metagenome-assembled genomes (MAGs) affiliated with other archaeal clades, including *Bathyarchaeota* (29), *Hadesarchaeota* (30), *Archaeoglobi* (30, 31), and “*Candidatus* Methanoliparia” (32). “*Ca.* Methanoliparia” archaea, previously known as the GoM-Arc2 clade (33), contain both canonical and divergent *mcr* genes (32). Therefore, it was suggested that “*Ca.* Methanoliparia” archaea may perform alkane degradation coupled with methanogenesis in a single organism (32), but this hypothesis lacks evidence by direct observation in cultures.

Sequences from “*Ca.* Syntrophoarchaeum,” “*Ca.* Argoarchaeum,” and “*Ca.* Methanoliparia” have been found in different oil and gas environments (34–38), including oil seeps from the Gulf of Mexico (33). Here, we studied sediment samples from such oil and asphalt seeps of the Campeche Knolls hydrocarbon province and analyzed them by combining geochemical methods, metagenomics, and fluorescence *in situ* hybridization (FISH). We hypothesize that naturally hydrocarbon-enriched, anoxic sediments contain diverse types of archaea specialized in the degradation of different alkanes and that “*Ca.* Methanoliparia” is associated with oil degradation to methane. Our main objective was to decipher the biochemical and genetic versatility of this and other types of archaea in the anaerobic degradation of hydrocarbons.

## RESULTS AND DISCUSSIONS

### Oil- and asphalt-rich sediments are inhabited by diverse bacterial and archaeal taxa involved in anaerobic alkane oxidation.

This study investigated hydrocarbon-rich marine sediments of the Campeche hydrocarbon province in the southern Gulf of Mexico for the presence of alkane-degrading archaea. We retrieved four samples with different biogeochemical signatures to test our hypothesis that specific types of archaea are responsible for the anaerobic degradation of different hydrocarbons (Table 1). The main sample used for this study, termed “oily sediment” (GeoB19351-14), was obtained from the surface of the seafloor in the vicinity of the Chapopote Knoll (see Fig. S1 in the supplemental material) (39, 40). These sediments contained large amounts of biodegraded heavy oil, as indicated by the absence of alkanes with carbon chain lengths of 12 to 20, and were sulfate depleted due to sulfate-dependent hydrocarbon degradation (Fig. S1A and B). Stable carbon isotopic compositions of methane indicate methanogenesis in those sediments (Fig. S1A and Text S1). The second sample (GeoB19345-1), here “asphalt flow,” was collected from the main asphalt field of the Chapopote Knoll (40). These freshly deposited asphalts do not show signs of strong biodegradation, and we consider them a reference for the original hydrocarbon composition (Text S1; Fig. S1B). A third sample (GeoB19351-5; here “ambient sediment”) was retrieved from nearby sediment with no exposure to oil or other hydrocarbons (Fig. S1D). The fourth sample (GeoB19331-1; here “asphalt sediment”) was retrieved using a gravity core from the Mictlan Knoll situated northeast of Chapopote (41, 42). The upper part of the core contained normal pelagic sediment. At its base (from an ∼135-cm sediment depth), solid asphalt pieces were retrieved to study their microbial communities.

**TABLE 1:** Summary of sample characteristics and analyses performed in this study

Sample name	GeoB no.^*a*^	Location	Sampling area	Sample description	Sampling core type (depth [cm])	Water depth (m)	Analysis(es)
Oily sediment	19351-14	21°53.964′N, 93°26.226′W	Chapopote	Oily sediment in the vicinity of the asphalt volcano	Push (1–10)	2,925	Geochemical analysis 16S rRNA gene tag sequencing, microscopy visualization, cell counting, metagenomics
Asphalt flow	19345‐1	21°53.964′N, 93°26.226′W	Chapopote	Solid and fresh asphalt from asphalt deposits	Gravity (40)	2,926	Geochemical analysis
Ambient sediment	19351-5	21°53.954′N, 93°26.261′W	Chapopote	Sediment not affected by oil or gas	Push (1–10)	2,905	16S rRNA gene tag sequencing, cell counting
Asphalt sediment	19331-1	22°01.354′N, 93°14.809′W	Mictlan	Sediment with solid asphalt pieces	Gravity (135)	3,092	16S rRNA gene tag sequencing, cell counting, metagenomics

a Permanent identifier.

10.1128/mBio.01814-19.1 TEXT S1 Supplemental text. (A) Supplementary Materials and Methods. (B) Supplementary Results and Discussion. Download Text S1, DOCX file, 0.03 MB. Copyright © 2019 Laso-Pérez et al. 2019 Laso-Pérez et al. This content is distributed under the terms of the Creative Commons Attribution 4.0 International license.

10.1128/mBio.01814-19.2 FIG S1 Geochemical analyses and sampling. (A) Geochemical analysis of oily sediment (GeoB19351-14). (Left) Concentrations of sulfate (black stars) and methane (red stars); (middle) concentrations of short-chain alkanes (triangles, ethane; squares, propane; diamonds, *i*-butane); (right) rates of sulfate reduction (black circles) and methane oxidation (red circles). pw, pore water; sed, sediment; d, day. (B) Analysis of the saturate fraction of the organic extract of oily sediment GeoB19351-14 at a sediment depth 5 to 7 cm below the sea floor (cmbsf) (top chromatogram) and a less biodegraded sample of the main asphalt field (GeoB19345-1; circa 45 cmbsf; bottom chromatogram) analyzed by gas chromatography at *m/z* 85. Note that the baseline in the oily sediment sample is elevated because most of the alkanes have been removed. UCM, unresolved complex mixture; EIC, extracted-ion chromatogram. (C) Sampling site of the oily sediment in the Chapopote Knoll. Push core sampling (GeoB19351-14) in a field covered by microbial mats, tube worms, and mussels in the vicinity of gas hydrates (not seen). (D) Sampling site of the ambient sediment (19351-5) at the Chapopote Knoll. Shown is a sediment elevation covered with a bacterial mat and tube worm bushes rooting in massive asphalt layers close by. Download FIG S1, PDF file, 2.4 MB. Copyright © 2019 Laso-Pérez et al. 2019 Laso-Pérez et al. This content is distributed under the terms of the Creative Commons Attribution 4.0 International license.

**FIG 1:**
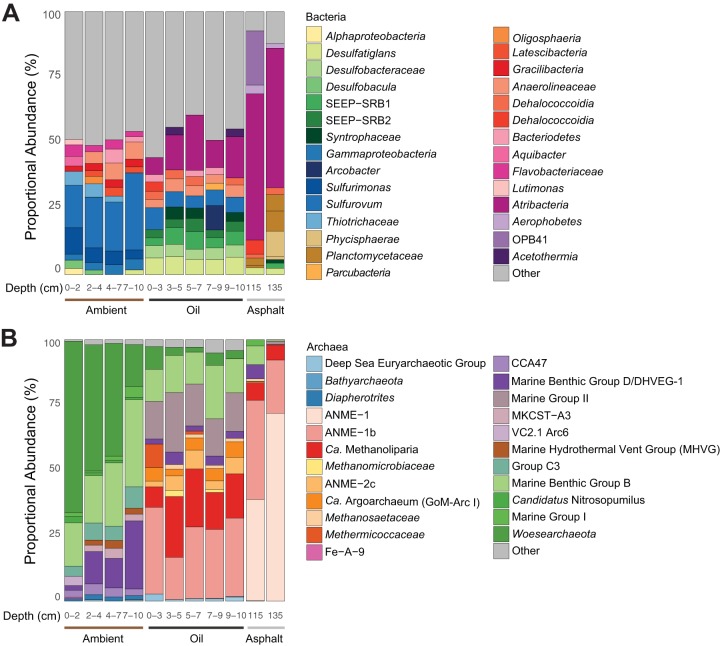
Relative abundances of 16S rRNA gene tags for bacteria (genus level) (A) and archaea (family level) (B) in the ambient, oily, and asphalt sediment. For each sample and depth, only the 10 most abundant clades are depicted. In the ambient sediment, the sequences of mostly aerobic and partly planktonic microorganisms were found. The oily site contained many archaeal sequences related to ANME-1, “*Ca.* Methanoliparia,” and “*Ca.* Argoarchaeum” (GoM-Arc1) as well as Deltaproteobacteria described as oil degraders (*Desulfobacula*, *Syntrophaceae*) and syntrophic partner bacteria of ANME (SEEP-SRB1). In the asphalt sediment, sequences of “*Ca.* Methanoliparia” were found only in the proximity of the asphalt pieces.

The sediment microbial communities of three of the sites (oily, ambient, and asphalt sediment) were characterized with bacterial and archaeal 16S rRNA gene amplicon libraries. The bacterial library of the oily sediment (Fig. 1A and Data Set S1A) contained mostly Deltaproteobacteria (23 to 32%) affiliated with *Desulfobacterales* (13 to 17%), *Desulfarculales* (6 to 7%), and *Syntrophobacterales* (3 to 5%). Some of them clustered within the clades Seep-SRB 1 and Seep-SRB 2, which include the partner bacteria of ANME in AOM (43, 44); others were suggested to be alkane degraders (8, 44–46). The archaeal libraries of the oily sediment (Fig. 1B and Data Set S1B) contained abundantly detected typical seep and deep-sea archaea, including ANME-1 (17 to 33%), marine benthic group B (*Thaumarchaeota*; 12 to 21%), ANME-2c (2 to 7%), and marine group II (14 to 23%), as well as the euryarchaeotal groups “*Ca.* Methanoliparia” (8 to 23%), “*Ca.* Argoarchaeum” (GoM-Arc1; 3 to 6%), and also “*Ca.* Syntrophoarchaeum” (<1%).

10.1128/mBio.01814-19.10 DATA SET S1 Relative abundances of the 16S rRNA gene tags and Methanoliparia_GoM genes involved in hydrocarbon metabolism. (A and B) Relative abundances of 16S rRNA gene tags in the ambient, oily, and asphalt sediment for bacteria (spreadsheet Bacterial 16S abundance) (A) and archaea (spreadsheet Archaeal 16S abundances) (B). (C to E) Overview of the Methanoliparia_GoM genes encoding enzymes for alkane degradation (spreadsheet Alkane_Degradation) (C), enzymes with the potential to be involved in the degradation of aromatic hydrocarbon genes (spreadsheet Aromatic_degradation) (D), and hydrogenases and other related proteins (spreadsheet Hydrogenases) (E). Download Data Set S1, XLSX file, 0.1 MB. Copyright © 2019 Laso-Pérez et al. 2019 Laso-Pérez et al. This content is distributed under the terms of the Creative Commons Attribution 4.0 International license.

In the asphalt sediment, bacterial 16S rRNA gene amplicon libraries contained mostly *Atribacteria* (53 to 65%) (Fig. 1A). The respective archaeal libraries were dominated by ANME-1 (77 to 92%) and contained considerable proportions of “*Ca.* Methanoliparia” (6 to 7%) (Fig. 1B). The libraries of the ambient sediment were different for both archaea and bacteria. The bacterial library (Data Set S1A) was dominated by *Epsilonproteobacteria* (24 to 33%) and *Gammaproteobacteria* (10 to 17%). *Thaumarchaeota* (24 to 47%) and “*Candidatus* Woesearchaeota” (15 to 65%) were the most abundant groups in the archaeal library. Alkane-degrading archaea like ANME, “*Ca.* Methanoliparia,” and “*Ca.* Argoarchaeum” were not detected at this site (Fig. 1B and Data Set S1B).

**FIG 2:**
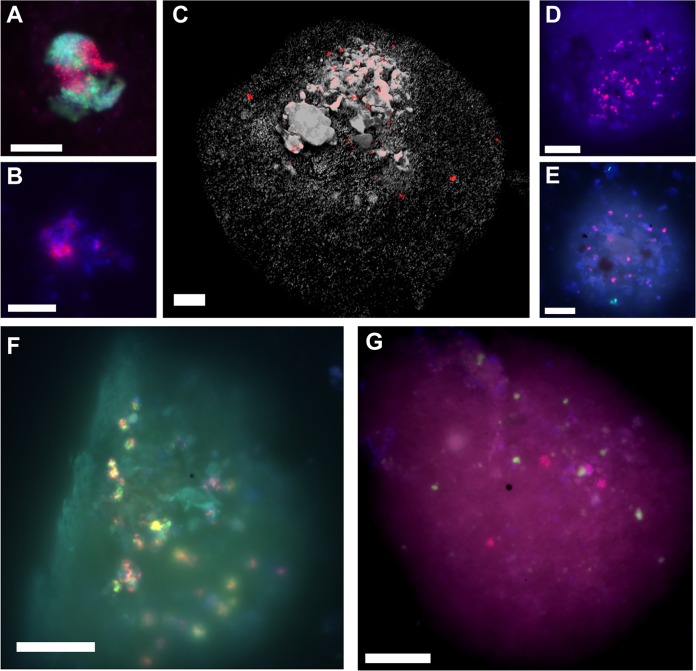
Epifluorescence micrographs of “*Ca.* Methanoliparia,” “*Ca.* Argoarchaeum” (GoM-Arc1), and “*Ca.* Syntrophoarchaeum” cells in oily sediment taken after CARD-FISH. (A to G) Visualizations of “*Ca.* Argoarchaeum” (GoM-Arc1) cells with the GOM-ARCI-660 probe (red) and bacteria (green) (A), “*Ca.* Syntrophoarchaeum” cells in consortia with the SYNA-666 probe (red) (B), “*Ca.* Methanoliparia” with the DC06-735 probe (red) in an immersed oil droplet (autofluorescence in gray) (C), “*Ca.* Methanoliparia” with the DC06-660 probe (red) in an immersed oil droplet (D), “*Ca.* Methanoliparia” cells (red) and bacteria (green; targeted with probe EUB388 I-III) (E), dual CARD-FISH staining of “*Ca.* Methanoliparia” cells with the specific probes DC06-735 (green) and DC06-660 (red) (F), and “*Ca.* Methanoliparia” cells (red, targeted with the DC06-735 probe) and ANME-1 cells (green) with the ANME-1-350 probe (G). (A, B, D, E, and F) Additional DAPI staining appears in blue. (D to G) Contours of oil droplets are visible by autofluorescence. Scale bars in all images, 10 μm.

Specific oligonucleotide probes (Materials and Methods; Table S1) targeting the 16S rRNA of specific microbial groups enabled the detection of “*Ca.* Syntrophoarchaeum” (up to 2.2% of archaeal cells or 6 × 10^6^ cells per ml sediment) and “*Ca.* Argoarchaeum” (up to 8.2% or 4 × 10^7^ cells per ml) in the oily sediment (Fig. S2B). Both organisms formed consortia with bacteria, as formerly reported (26, 28) (Fig. 2A and B). Notably, all sampled horizons of the oily sediment contained high numbers of “*Ca.* Methanoliparia” organisms, with estimated abundances between 7 × 10^6^ and 1 × 10^7^ cells per ml of oily sediment (Fig. S2B). These archaea were found associated primarily with oil droplets and appear as single cells or as multicellular chains (Fig. 2C to G). The oil scattered and absorbed light, and hence only cells close to the surface droplet could be visualized. Therefore, calculated cell numbers for “*Ca.* Methanoliparia” likely underestimate the true abundance of this group. No bacterial or ANME-1 cells were detected associated with “*Ca.* Methanoliparia” cells, suggesting that “*Ca.* Methanoliparia” does not form consortia and may be independent from syntrophic associations with other microorganisms (Fig. 2E and G). In the asphalt sediment, only cells of “*Ca.* Argoarchaeum” were found (Fig. S2B). None of the three target groups was detected in the ambient sediment (Fig. S2B).

10.1128/mBio.01814-19.3 FIG S2 Relative and absolute abundances of microorganisms. (A) Relative abundances of different microbial families based on the analysis of 16S rRNA gene fragments from the metagenomic libraries performed with phyloFlash; (B) microscopic counts of different microbial clades based on CARD-FISH counts. Estimated cell abundances for *Bacteria* and *Archaea* and for the clades “*Ca.* Methanoliparia,” “*Ca.* Argoarchaeum” and “*Ca.* Syntrophoarchaeum” are shown. Cell abundances are shown on a logarithmic scale. Bars below the axis indicate that no cells were found in the sample. Download FIG S2, PDF file, 0.4 MB. Copyright © 2019 Laso-Pérez et al. 2019 Laso-Pérez et al. This content is distributed under the terms of the Creative Commons Attribution 4.0 International license.

10.1128/mBio.01814-19.6 TABLE S1 CARD-FISH probes, competitors, and helpers applied in this study and primers used for the PCR sequencing of the 16S rRNA gene amplicon libraries prior to Illumina sequencing. FA%, formamide concentration. Download Table S1, DOCX file, 0.02 MB. Copyright © 2019 Laso-Pérez et al. 2019 Laso-Pérez et al. This content is distributed under the terms of the Creative Commons Attribution 4.0 International license.

### Metagenomic sequencing and phylogenetic analysis of assembled genomes.

To investigate the metabolic potential of the targeted archaea, total DNA metagenomic libraries were prepared and sequenced from the oily sediment and the asphalt sediment (Table S2). The phylogenetic compositions of these libraries were assessed based on the 16S rRNA gene fragments using phyloFlash (Fig. S2A). In the libraries of the oily sediment, 82 to 89% of the 16S rRNA gene fragments were affiliated with bacteria and 11 to 18% with archaea. “*Ca.* Methanoliparia” represented between 20 and 30% of the archaeal community and between 2 and 5% of the total community. In the asphalt sediment library, archaea were more dominant than bacteria (78% versus 22%). In this sample, most of the fragments were affiliated with the ANME-1 clade (73%), but a small fraction (5%) belonged to “*Ca.* Methanoliparia.”

10.1128/mBio.01814-19.7 TABLE S2 Metagenomic libraries used for the assembly of Methanoliparia_GoM MAGs. Download Table S2, DOCX file, 0.01 MB. Copyright © 2019 Laso-Pérez et al. 2019 Laso-Pérez et al. This content is distributed under the terms of the Creative Commons Attribution 4.0 International license.

The metagenomic reads of each sample were assembled, and contigs were binned. This yielded two high-quality metagenome assembled genomes (MAGs) from the oily and the asphalt sediment, which contained a full-length 16S rRNA gene affiliated with “*Ca.* Methanoliparia.” We named the new MAGs Methanoliparia_GoM_oil, i.e., the MAG obtained from the oily sediment, and Methanoliparia_GoM_asphalt, i.e., the MAG obtained from the asphalt sediment. The two MAGs have sizes of 1.70 and 1.75 Mb, with estimated completeness percentages of 75% and 92% and a contamination of less than 2% (Table S3). They have a high average nucleotide identity (ANI) to each other (ANI = ∼93 to 95%) (Table S3); hence, they likely belong to the same species according to genomic standards (47). These two MAGs have as well a high average nucleotide identity to the genome of the previously described “*Candidatus* Methanolliviera hydrocarbonicum” (∼92 to 94%) (Table S3) (32), indicating that the MAGs retrieved in this study belong at least to a closely related species.

10.1128/mBio.01814-19.8 TABLE S3 Genomic information from the extracted MAGs (Methanoliparia_GoM_oil and Methanoliparia_GoM_asphalt) and Syntropho_SAG affiliated with “*Ca.* Syntrophoarchaeum” and a pairwise whole-genome identity comparison between Methanoliparia_GoM MAGs and the genomes of “*Ca.* Methanolliviera hydrocarbonicum” and “*Candidatus* Methanolliviera thermophilum” and between Syntropho_SAG and the genomes of “*Candidatus* Syntrophoarchaeum butanivorans” and “*Candidatus* Syntrophoarchaeum caldarius.” ANI, average nucleotide identity. Download Table S3, DOCX file, 0.02 MB. Copyright © 2019 Laso-Pérez et al. 2019 Laso-Pérez et al. This content is distributed under the terms of the Creative Commons Attribution 4.0 International license.

MAGs of “*Ca.* Argoarchaeum” and “*Ca.* Syntrophoarchaeum” were not retrieved. However, we were able to obtain a single amplified genome of “*Ca.* Syntrophoarchaeum” based on single-cell sorting followed by whole genome amplification and sequencing. The retrieved single amplified genome (SAG) (Syntropho_SAG) had a size of 0.81 Mb and a completeness of 42.7% (Table S3). It likely represents a new species within the genus “*Ca.* Syntrophoarchaeum” (Text S1). The limited genomic information shows a metabolic potential similar to that of the cultured species of “*Ca.* Syntrophoarchaeum” (Text S1; Fig. S3) (26). Cells affiliated with “*Ca.* Methanoliparia” could not be sorted, likely because these archaea are associated with the oil droplets.

10.1128/mBio.01814-19.4 FIG S3 Enzymes encoded in the genomes of Syntropho_SAG based on the metabolic model of “*Ca.* Syntrophoarchaeum” (R. Laso-Pérez, G. Wegener, K. Knittel, F. Widdel, K. J. Harding, et al., Nature 539:396, 2016, https://doi.org/10.1038/nature20152). The genes encoding the corresponding enzymes are shown in color: red for alkane activation/reverse methanogenesis, blue for the fatty acid oxidation pathway, and purple for the Met complex. In gray are steps of the metabolic model of “*Ca.* Syntrophoarchaeum,” for which no gene was found in Syntropho_SAG. Download FIG S3, EPS file, 1.4 MB. Copyright © 2019 Laso-Pérez et al. 2019 Laso-Pérez et al. This content is distributed under the terms of the Creative Commons Attribution 4.0 International license.

**FIG 3:**
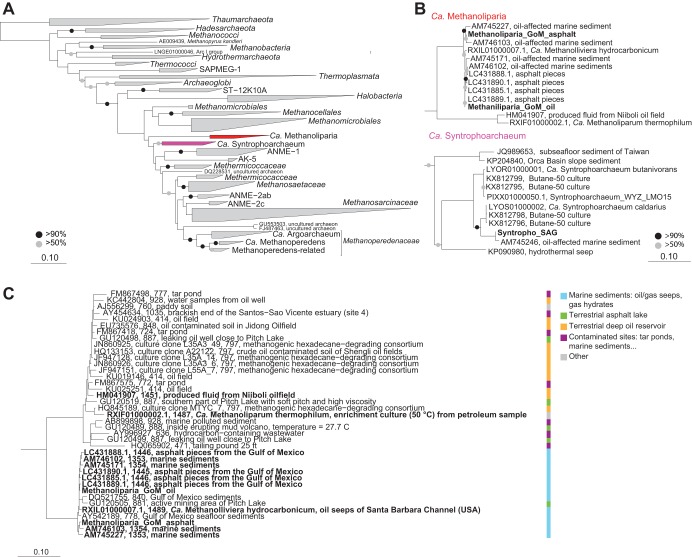
Phylogenetic affiliation of “*Ca.* Syntrophoarchaeum” and “*Ca.* Methanoliparia” based on the 16S rRNA gene. (A) Phylogenetic tree of the 16S rRNA gene of the phylum *Euryarchaeota*, with a focus on *Methanomicrobia*. Sequences of the “*Ca.* Methanoliparia” clade are shown in red, and sequences of the “*Ca.* Syntrophoarchaeum” clade are shown in purple. (B) Extended display of the clades of interest, with 16S rRNA full-length gene sequences of the “*Ca.* Methanoliparia” and “*Ca.* Syntrophoarchaeum” bins in bold. (C) Phylogeny of the “*Ca.* Methanoliparia” clade based on the 16S rRNA gene, including full-length sequences (in bold) and representative short sequences from studies that detected “*Ca.* Methanoliparia” in SILVA database v132. The tags refer to accession numbers, clone identifiers (when applicable), lengths, and isolation origins. On the right, the classifications of sequences according to their environmental origins are shown. For all panels, scale bars represent the number of nucleotide substitutions per site. For panels A and B, bootstrap values over 50% and 90% are indicated with gray and black circles, respectively.

Both Methanoliparia_GoM MAGs and the Syntropho_SAG contained complete 16S rRNA genes that were used to construct a comprehensive phylogenetic tree, including all sequences of the target clades longer than 1,350 bp present in the SILVA database (Fig. 3A). “*Ca.* Syntrophoarchaeum” sequences form a distinct clade within *Methanomicrobia*, likely representing a separate order (Text S1). “*Ca.* Methanoliparia” sequences cluster in a group close to *Methanomicrobia.* These sequences exclusively derive from sites with liquid alkanes, such as marine oil seeps or terrestrial oil reservoirs, and include the 16S rRNA gene sequences from our two “*Ca.* Methanoliparia” MAGs (Fig. 3B; Table S4). Interestingly, a 16S rRNA gene phylogeny of the “*Ca.* Methanoliparia” clade including additional short sequences, reveals the existence of two subgroups within the clade (Fig. 3C). The two 16S rRNA gene sequences from our MAGs form a cluster with other sequences from marine oil containing environments, while a second group includes sequences from terrestrial oil reservoirs, asphalt lakes, and contaminated sites. With values of 78 to 82% identity to the sister clades, “*Ca.* Methanoliparia” likely represents a distinct class within *Euryarchaeota*.

10.1128/mBio.01814-19.9 TABLE S4 Representative accession numbers and environmental information from publicly available 16S rRNA gene sequences affiliated with “*Ca.* Methanoliparia” from the SILVA v132 database. Sequence lengths range from 394 to 1.451 bp. mbsl, meters below sea level. Download Table S4, DOCX file, 0.02 MB. Copyright © 2019 Laso-Pérez et al. 2019 Laso-Pérez et al. This content is distributed under the terms of the Creative Commons Attribution 4.0 International license.

### Genomic metabolic potential of Methanoliparia_GoM MAGs.

**FIG 4:**
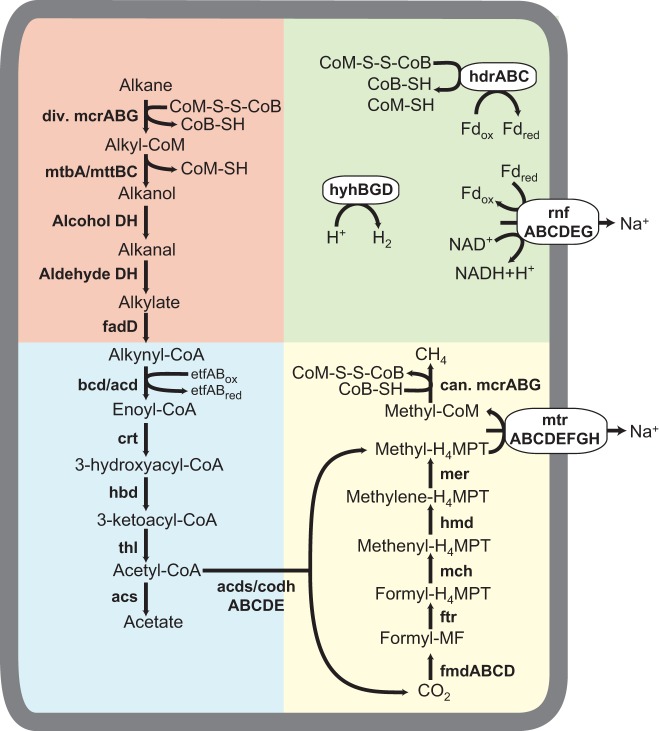
Metabolic model of alkane disproportionation proposed for Methanoliparia_GoM. Genes for all the proposed steps were found in the MAGs. Background colors indicate alkane activation and conversion to a CoA-bound fatty acid (red), fatty acid degradation and the ACDS complex (blue), methanogenesis (yellow), and electron cycling (green). The alkanes are activated by forming alkyl-CoM as the primary intermediate by the divergent (div.) MCR. Then, free alcohols form and are sequentially oxidized to fatty acids, which are ligated to CoA and oxidized into acetyl-CoA. This molecule is split into CO_2_ and H_4_MPT-bound methyl groups. Methyl-H_4_MPT is reduced to methane with the canonical (can.) MCR. The surplus reducing equivalents released during this alkane disproportionation are used for additional methane formation through CO_2_ reduction. Additional energy-conserving steps are possible via the regeneration of CoM-S-S-CoB and the transfer of electrons from ferredoxin to NADH. A hydrogenase may be present to balance additional electron gradients.

**FIG 5:**
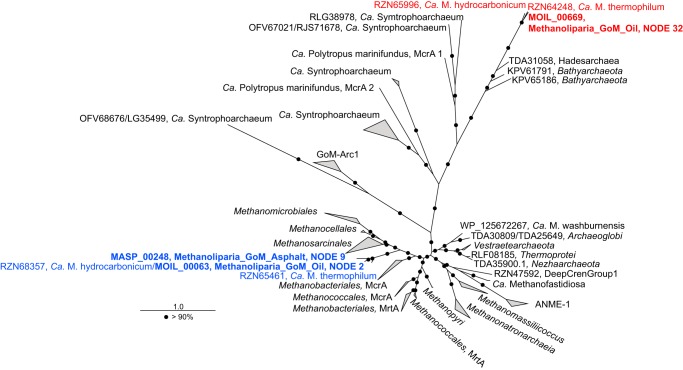
Phylogenetic affiliation of amino acid sequences of the *mcrA* genes present in Methanoliparia_GoM MAGs. The tree was calculated based on a maximum-likelihood algorithm using 305 amino acid sequences using over 450 amino acid positions for calculations. “*Ca.* Methanoliparia” *mcrA* genes are indicated in red (related to the divergent *mcr* genes of *Bathyarchaeota*, *Hadesarchaea*, and “*Ca.* Polytropus marinifundus”) or blue (related to methane cycling) and indicate the locus tag, original MAG, and scaffold. The inclusion of the divergent McrA of Methanoliparia_GoM_asphalt in this phylogenetic analysis was not possible, as the corresponding *mcrA* sequence was truncated and could not be recovered since the *mcr* operon was located in a short contig. The scale bar indicates the number of amino acid substitutions per site. Bootstrap values of >90% are indicated with black circles.

Both MAGs retrieved in this study, Methanoliparia_GoM_oil and Methanoliparia_GoM_asphalt, encode for identical metabolic potentials (Fig. 4; Data Set S1C). Therefore, they are discussed together as Methanoliparia_GoM. Similarly, the genomic metabolic potential is comparable to that of the previously described “*Ca.* Methanoliparia” genomes (32). Most remarkable is that Methanoliparia_GoM also encodes two MCRs: one canonical and one divergent, related to the alkane-degrading MCRs (Fig. 5; Fig. S4). Consequently, Methanoliparia_GoM might have the potential to activate multicarbon alkanes but also to produce or oxidize methane.

10.1128/mBio.01814-19.5 FIG S4 Alignment of regions with catalytically relevant residues of MCR-A. Residues are numbered according to the system of Ermler et al. (U. Ermler, W. Grabarse, S. Shima, M. Goubeaud, and R. K. Thauer, Science 278:1457–1462, 1997, https://doi.org/10.1126/science.278.5342.1457). Amino acids are colored based on their chemical features using the software Seaview (M. Gouy, S. Guindon, and O. Gascuel, Mol Biol Evol 27:221–224, 2010, https://doi.org/10.1093/molbev/msp259). The alignment includes a selection of representative novel alkane-degrading MCRs (red box) and canonical MCRs of methanogens and ANME (blue box). Important residues interacting with elements of the catalysis or with posttranslational modifications are indicated under the alignment. MCR-As of Methanoliparia_GoM are included in both boxes and indicated with a bold name. Download FIG S4, PDF file, 2.9 MB. Copyright © 2019 Laso-Pérez et al. 2019 Laso-Pérez et al. This content is distributed under the terms of the Creative Commons Attribution 4.0 International license.

**FIG 6:**
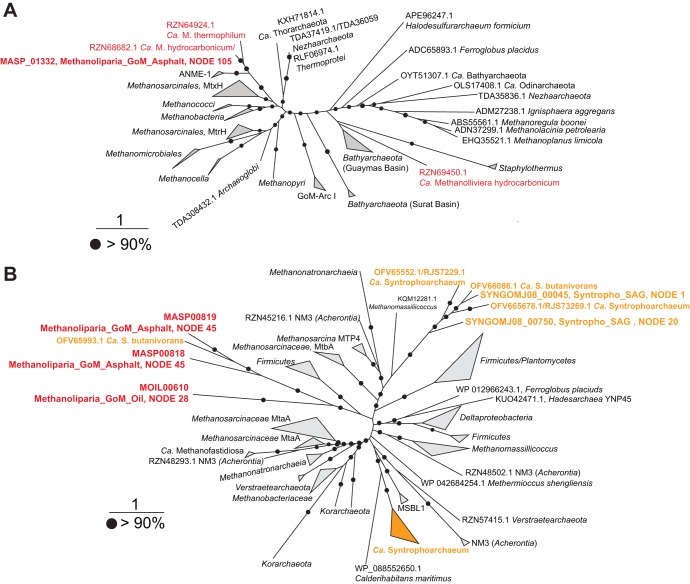
Phylogenetic affiliations of the amino acid sequences of the *mtrH* (A) and *mta* (B) genes present in the Methanoliparia_GoM and “*Ca.* Syntrophoarchaeum” bins. (A) Phylogenetic tree of the amino acid sequence of the *mtrH* gene. The sequence present in the Methanoliparia_GoM_asphalt MAG is red. An *mtrH* gene was found neither in Methanoliparia_GoM_oil nor in Syntropho_SAG. (B) Phylogenetic tree of the amino acid sequence of the *mta* genes. The sequence present in Methanoliparia_GoM is red. “*Ca.* Syntrophoarchaeum” sequences, including those found in Syntropho_SAG and in previous studies, are orange. For both trees, scale bars represent the number of amino acid substitutions per site, and black circles represent bootstrap values of over 90%. The name includes the locus tag of the gene, the MAG or SAG, and the corresponding node.

**(i) Methanogenesis pathway.** According to phylogenetic analysis, the canonical McrA of Methanoliparia_GoM forms an independent cluster related to McrA sequences of *Methanomicrobia* and *Methanobacteriales* (Fig. 5). Additionally to the canonical MCR, all genes encoding the other enzymes of the methanogenesis pathway are present in Methanoliparia_GoM (Fig. 4; Data Set S1C), including the methyl-tetrahydromethanopterin (H_4_MPT):coenzyme M methyltransferase (Mtr) and the *N*^5^,*N*^10^-methylene tetrahydromethanopterin reductase (Mer). In methanogens and anaerobic methanotrophs, Mtr catalyzes the transfer of a methyl radical between CoM and tetrahydromethanopterin. A phylogenetic analysis of the catalytic subunit MtrH shows a close affiliation of the Mtr of Methanoliparia_GoM with those of methanogens (*Methanosarcinales*) and methanotrophs (ANME-1) (Fig. 6A). This suggests that the Mtr of Methanoliparia_GoM catalyzes the transfer of C_1_ compounds. Therefore, it is part of an intact methanogenesis pathway. In contrast, the Syntropho_SAG of this study and the previously published genomes of “*Ca.* Syntrophoarchaeum” do not contain *mtr* genes. These organisms do not require Mtr, since they oxidize the alkane-derived methyl units (26).

**(ii) Alkane activation by MCR.** The other *mcr* operon, present in both MAGs of Methanoliparia_GoM, contains a highly divergent *mcrA*, which shares only 41% identity with the canonical *mcrA* at the protein level but has high identity to the published divergent MCRs of “*Ca.* Methanoliparia” (83 to 86%) (32), which are closely affiliated with the divergent McrA types found in *Bathyarchaeota* (29), *Hadesarchaea* (30), and “*Candidatus* Polytropus marinifundus” (*Archaeoglobi*) (31) (Fig. 5). These divergent McrA types are related to those of “*Ca.* Syntrophoarchaeum,” which activate multicarbon alkanes, such as butane and propane (26), forming alkyl-CoM as a primary intermediate.

Additionally, Methanoliparia_GoM harbors genes encoding several methylcobalamin:coenzyme M methyltransferases (related to MtaA and MtbA/MttBC) (Data Set S1C). In “*Ca.* Syntrophoarchaeum,” these enzymes were suggested to transform the butyl-CoM into an intermediate, which after oxidation is converted to butyryl-CoA (26). The cobalamin methyltransferase genes from Methanoliparia_GoM clusters in a deeply branching clade with one of the *mtaA* copies of “*Ca.* Syntrophoarchaeum” (Fig. 6B), suggesting a similar mechanism for the initial transformation of CoM-bound alkanes in Methanoliparia_GoM. Moreover, the Methanoliparia_GoM MAGs harbor genes that may encode the following steps toward a CoA-bound fatty acid (Fig. 4; Data Set S1C): the CoM-bound alkyl units may be released as free alcohol using the encoded subunit B of the cobalamin methyltransferase. The released alcohol might then be sequentially oxidized by an alcohol and an aldehyde dehydrogenase, which are encoded in the MAGs. The produced fatty acid might be ligated to CoA using the encoded CoA ligases. Interestingly, the Methanoliparia_GoM MAGs encode several long-chain fatty-acid–CoA ligases (*fadD*) and other CoA transferases, which may indicate the potential to degrade a variety of different long- and short-chain alkanes. Genes encoding FadD have also been found in “*Ca.* Polytropus marinifundus” (31) and the other “*Ca.* Methanoliparia” organisms (32), but they were thought to degrade long-chain carboxylic acids. This proposed link between CoM-bound alkyl units and the CoA-bound fatty acid was not found in the genomes of “*Ca.* Syntrophoarchaeum” organisms, which lack these CoA ligases (26). This may indicate that “*Ca.* Syntrophoarchaeum” and Methanoliparia_GoM use different strategies to connect the CoM activation with the fatty acid degradation.

**(iii) Fatty acid degradation and the Wood-Ljungdahl pathway.** Methanoliparia_GoM encodes a complete fatty acid degradation pathway, of which many steps are encoded in several copies per single MAG (Fig. 4; Data Set S1C). This allows the oxidation of diverse CoA-bound fatty acids into acetyl-CoA as shown for “*Ca.* Syntrophoarchaeum” (26). Several encoded enzymes in the MAGs may metabolize the acetyl-CoA, including the acetyl-CoA decarbonylase/synthase (ACDS) complex and acetyl-CoA synthetase. The ACDS complex should work in the oxidative direction, cleaving the acetyl-CoA into CO_2_ and methyl-H_4_MPT. For “*Ca.* Syntrophoarchaeum,” it has been suggested that the H_4_MPT-bound methyl groups are fully oxidized with the downstream part of the methanogenesis pathway (26). However, the presence of the *mtr* and two distinct *mcr* operons in Methanoliparia_GoM may indicate an alternative route of this methyl group, toward the production of methane (32).

Interestingly, Methanoliparia_GoM may also have the potential for the degradation of aromatic compounds like benzene (Data Set S1D). Both MAGs contain an operon encoding two subunits of the benzoyl-CoA reductase (BadEG) and methylmalonyl-CoA mutases. The Methanoliparia_GoM_oil operon includes a gene encoding a CoA ligase, whereas the Methanoliparia_GoM_asphalt operon contains another gene encoding an additional benzoyl-CoA reductase subunit (BadD). However, these *badDEG* genes also have high similarities with the genes encoding 2-hydroxymethylglutaryl-CoA dehydratase. This enzyme is involved in the degradation of l-glutamate. Thus, the identified *bad* genes may encode alternative functions. Genes encoding enzymes for the steps of benzoyl-CoA degradation (48), like cyclic dienoyl-CoA hydratase or dienoyl-CoA-reducing enzymes, have not been detected, although some are similar to enzymes of the fatty acid degradation pathway. Products and reducing equivalents of the degradation of aromatic compounds may be similarly used for the production of methane. Further studies are needed to elucidate the potential of Methanoliparia_GoM to degrade alkanes and aromatic compounds.

**(iv) Electron cycling.** Genes encoding several electron cycling complexes are present in both MAGs, including the complex Rnf, the CoB-CoM heterodisulfide reductase (HdrABC), several 4Fe-4S proteins associated with aldehydes oxidoreductases (Data Set S1C), and a hydrogenase (Hyh), related to those of Pyrococcus furiosus (Data Set S1E). The Hyh enzymes of P. furiosus are formed by four subunits (49, 50). However, only the beta and gamma subunits were present in both Methanoliparia_GoM MAGs, while the alpha catalytic subunit was absent. This absence may be attributed to the incompleteness of the MAGs. Alternatively, the Hyh protein of Methanoliparia_GoM may not be involved in hydrogen metabolism but only in electron cycling. Associated with the *hdr* operon, the Methanoliparia_GoM MAGs contain electron transfer flavoproteins and an Fe-S oxidoreductase, which are probably involved in the electron flow during fatty acid oxidation (51). Additionally, the Methanoliparia_GoM_oil MAG has an operon encoding several hydrogenase subunits resembling the ferredoxin hydrogenase (Mvh) and a NiFe hydrogenase (Data Set S1E), which are thought to be involved in CoM regeneration in different methanogens (50). As with the previously described “*Ca.* Methanoliparia” species, Methanoliparia_GoM MAGs likely lack multiheme *c-*type cytochromes involved in direct electron transfer (32). The encoded cytochromes present in Methanoliparia_GoM are associated with genes involved in the detoxification of reactive oxygen species. In syntrophic alkane oxidizers, such as ANME-1, ANME-2, “*Ca.* Argoarchaeum,” and “*Ca.* Syntrophoarchaeum,” cytoplasmic and extracellular cytochromes are abundant and are thought to mediate direct electron transfer (15, 26, 28, 52, 53). This supports the hypothesis that “*Ca.* Methanoliparia,” which we observed exclusively as single cells in oily environments (Fig. 2), does not depend on syntrophic electron transfer.

The genomic information alone appears insufficient to predict the interplay and direction of the encoded enzymes. However, our analyses show that Methanoliparia_GoM has the machinery required for internal electron cycling and energy conservation. As with “*Ca.* Syntrophoarchaeum” and ANME, Methanoliparia_GoM lacks a dissimilatory sulfate reduction pathway. Methanoliparia_GoM_oil harbors genes encoding the phosphoadenosine phosphosulfate reductase, an adenylyl-sulfate kinase, and a sulfate adenylyltransferase, but these genes encode enzymes for assimilatory purposes.

### Metabolic model for Methanoliparia_GoM.

Based on the genomic composition and the environmental observations, we developed a preliminary metabolic model of the target organism, Methanoliparia_GoM. The phylogenetic analysis of the detected divergent MCR type suggests that Methanoliparia_GoM can activate alkanes, forming a CoM-bound alkyl unit as primary intermediate. This CoM-bound alkyl unit would be further degraded to CO_2_ and methyl-H_4_MPT (Fig. 4). Two possibilities emerge for the role of the canonical MCR. Either Methanoliparia_GoM organisms thrive on complete oxidation of methane and/or hydrocarbons or they disproportionate alkanes, producing methane and CO_2_ as final products. In the first scenario, Methanoliparia_GoM would use both of its MCRs in the oxidative direction, defining Methanoliparia_GoM as a novel ANME clade with the additional ability to metabolize higher hydrocarbons. It would fully oxidize the methane- and alkane-derived methyl-H_4_MPT with the downstream part of the reverse methanogenesis. However, the released reducing equivalents would need a sink. Methanoliparia_GoM lacks a bacterial partner and does not encode a dissimilatory sulfate reduction pathway or multiheme *c* cytochromes involved in direct electron transfer. In principle, reducing equivalents might be used to form hydrogen or acetate, but under standard conditions, such reactions are highly endergonic (Table 2). Extremely low *in situ* intermediate concentrations would need to be maintained, and there are currently no indications for such a scenario.

**TABLE 2:**
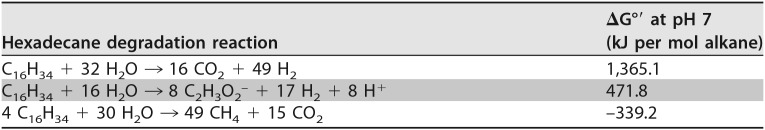
Thermodynamic calculations for the degradation of hexadecane under different conditions^*a*^

Hexadecane degradation reaction	ΔG°′ at pH 7 (kJ per mol alkane)
C_16_H_34_ + 32 H_2_O → 16 CO_2_ + 49 H_2_	1,365.1
C_16_H_34_ + 16 H_2_O → 8 C_2_H_3_O_2_^–^ + 17 H_2_ + 8 H^+^	471.8
4 C_16_H_34_ + 30 H_2_O → 49 CH_4_ + 15 CO_2_	–339.2

a Complete oxidation, oxidation to acetate, and oxidation coupled with methane formation.

In the second scenario, Methanoliparia_GoM would degrade alkanes and produce methane and CO_2_. In this case, the Mtr of Methanoliparia_GoM would transfer the methyl group from H_4_MPT to CoM, forming methyl-CoM, which could be finally reduced to methane using the canonical MCR. The surplus reducing equivalents released during the cleavage would be used for the reduction of CO_2_, resulting in additional methane (Fig. 4). A similar mechanism was proposed for the closely related “*Ca.* Methanoliparia” MAGs previously described (32).

On the basis of metagenomic data, we cannot define the range of alkanes that Methanoliparia_GoM may metabolize. Under standard conditions, methanogenic alkane degradation would be exergonic for all multicarbon alkanes (54), and energy yields would increase with alkane chain length (55). However, experiments with environmental samples showed alkane-dependent methanogenesis only for substrates with at least 6 carbon atoms (56). To date, CoM-type activation of hydrocarbons has been demonstrated only for short-chain alkanes up to *n-*butane (26, 28). Interestingly, the MAGs of Methanoliparia_GoM encode several long-chain acyl-CoA synthetases (Data Set S1C). This suggests that in Methanoliparia_GoM, divergent MCRs may catalyze the activation of mid- or long-chain alkanes coupled to methanogenesis. Notably, the oily sediment was almost completely depleted in alkanes with fewer than 20 carbon atoms (Fig. S1B; Text S1). Therefore, we hypothesize that Methanoliparia_GoM can degrade alkane to methane and discuss this process with hexadecane as an example, one of the most abundant compounds in the saturated fraction of the non-biodegraded asphalt flow. Without considering biosynthesis, the overall catabolic reaction for four hexadecane molecules would be as follows:

$$4\;{\text{C}}_{\text{16}}{\text{H}}_{\text{34}}\text{ + 30 }{\text{H}}_{\text{2}}\text{O}\to \text{49 }{\text{CH}}_{\text{4}}\text{ + 15}{\text{ CO}}_{\text{2}}$$

This reaction is exergonic under standard conditions (Table 2) but also under a wide range of environmental conditions, including those in the sampled oily deep-sea environment. The ability of microbial consortia to methanogenically degrade hydrocarbons has already been described, but so far, it has been thought to rely on the syntrophic interaction of hydrocarbon-degrading bacteria with methanogenic archaea (24, 25, 57). Methanoliparia_GoM does not form apparent associations with other partner organisms and lacks essential genes for syntrophic electron transfer; hence, we propose that the complete reaction may proceed in a single cell. In a first step, the alkane oxidation (Fig. 4) would produce methyl groups bound to H_4_MPT (represented as R-) and CO_2_ in equal amounts, releasing 200 electrons (represented as e^−^).

$$\text{4 }{\text{C}}_{\text{16}}{\text{H}}_{\text{34}}\text{ + 64}{\text{ H}}_{\text{2}}\text{O + 32 R-H}\to \text{32 R−}{\text{CH}}_{\text{3}}\text{ + 32 }{\text{CO}}_{\text{2}}\text{ + 200}{\text{ H}}^{\text{+}}\text{ + }{\text{200e}}^{-}$$

Of these electrons, 64 would be used to reduce the methyl-H_4_MPT to methane in an energy-conserving step, through the translocation of sodium ions out of the membrane.

$$32\text{ R−}{\text{CH}}_{3}\;+\;64{\text{e}}^{-}\;+\;64{\text{H}}^{+}\to 32{\text{ CH}}_{\text{4}}\;+\;32\text{ R-H}$$

The remaining 136 electrons would need an electron sink, likely in the form of methanogenic CO_2_ reduction.

$$17{\text{ CO}}_{2}\;+\;136{\text{H}}^{+}+136{\text{e}}^{-}\to 17\;{\text{CH}}_{4}\;+\;34\;{\text{H}}_{2}\text{O}$$

### Environmental data support alkane-dependent methanogenesis by Methanoliparia_GoM.

In this study, we investigated two MAGs of “*Ca.* Methanoliparia,” a clade of yet-uncultured anaerobic archaea that inhabit oil-rich and mostly sulfate-depleted environments (Table S4) (33, 58, 59). Its metabolic potential suggests that Methanoliparia_GoM archaea are able to disproportionate alkanes, producing methane and CO_2_ as final products in a single cell, as already suggested for other members of “*Ca.* Methanoliparia” (32). Microscopic observation of Methanoliparia_GoM cells in oily sediment from the Gulf of Mexico showed that these archaea are associated with oil droplets and lack partner organisms (Fig. 2C to F). Methanoliparia_GoM was evenly abundant throughout the sediment core, including the sulfate-depleted horizons (Fig. S2B). In the sediment samples, methane was slightly depleted in ^13^C (δ^13^C-CH_4_, −54.4 to –54.5‰) (Fig. S1A) compared to methane in a sample of gas bubbles escaping the seafloor (δ^13^C-CH_4_ = −46.5‰) that is assumed to reflect a higher proportion of thermogenic gases (60). This depletion in ^13^C points toward biological methanogenesis in these shallow sediments. Methanoliparia_GoM may be the key methane producer in the oily sediment based on its high proportional abundance (Fig. 1B; Fig. S2A), especially considering that canonical methanogens are less abundant in this environment (from 3% of the archaeal community in the deeper sediment to 9.6% in the shallower depths).

Gene sequences encoding the 16S rRNA of “*Ca.* Methanoliparia” have been detected in deep petroleum reservoirs and oil seeps (Table S4). These environments are rather neutral in pH and include cold and moderately heated environments. Notably, they were also abundant in methanogenic enrichment cultures from oily sediment (59, 61). However, they are only rarely found at gas seeps (Table S4). As mentioned above, the “*Ca.* Methanoliparia” clade contains two subgroups according to the 16S rRNA gene phylogeny (Fig. 3C): one of 16S rRNA sequences retrieved from marine oil environments and a second one with sequences from terrestrial oil reservoirs, asphalt lakes, and contaminated sites. The second group also includes short sequences of “*Ca.* Methanoliparia” detected in a hexadecane-degrading enrichment that were established from production water of a terrestrial oil reservoir (61) (Fig. 3C). Moreover, “*Ca.* Methanoliparia” was detected in methanogenic enrichments degrading crude oil established from oil field sludge at 35 and 55°C (59). That study assumed that the consumption of alkanes in the enrichments was performed by consortia of alkane-degrading bacteria and methanogenic archaea. However, our results suggest that “*Ca.* Methanoliparia” may be a key player in methanogenic oil degradation, without the need of a syntrophic partner. This implies a hitherto-overlooked role of archaea in hydrocarbon degradation. In particular, these archaea might be responsible for the transformation of alkane to methane in deep petroleum reservoirs. The cultivation of “*Ca.* Methanoliparia” will be a necessary future step to confirm this hypothesis.

## MATERIALS AND METHODS

### Sampling of hydrocarbon-rich sediments.

Sediment samples were collected during *RV Meteor* cruise M114-2 in March 2015 at Campeche Knolls in the Gulf of Mexico (42) (Table 1). Sediment samples from the targeted gas and oil seeps (oily sediment, push core GeoB19351-14) were collected at the Chapopote Knoll by push coring with the *ROV MARUM-Quest*. The solid and fresh asphalt flow (40-cm deep) was sampled in the vicinity by gravity coring (GeoB19345-1). A sediment sample without any oil or asphalt (ambient sediment, GeoB19351-5) was also retrieved from a push coring dive from a nearby location at the rim of the main asphalt field. The asphaltic sediment sample (asphalt sediment, GeoB19331-1) was retrieved from the Mictlan Knoll using a gravity corer. On-board sediments were sectioned and fixed for different methods, including cell counting, fluorescence *in situ* hybridization, nucleic acid extraction, and geochemical analysis.

### Geochemical measurements.

Concentrations of short-chain alkanes (C_1_ to *n-*C_6_) in samples from push core GeoB19351-14 were determined on board by gas chromatography and flame ionization detection (62, 91). Methane stable carbon isotopic compositions were measured in the home lab using isotope ratio mass spectrometry. Rates of methane oxidation and sulfate reduction were determined by incubations with [^14^C]methane and [^35^S]sulfate, by separate analysis of reactant (methane, sulfate) concentrations, and by determination of radioactivity in the reactant and product (inorganic carbon, total reduced inorganic sulfur) as described in references 63 and 64. To determine the distribution patterns of long-chain hydrocarbons in oily sediment (GeoB19351-14) and asphalt flow (GeoB19345-1), the organic fractions in those samples were extracted with dichloromethane (DCM), and after asphaltene precipitation, the *n*-hexane fractions were separated via silica gel column chromatography with *n*-hexane into saturate fractions. These fractions were analyzed by gas chromatography coupled to mass spectrometry, with the spectrometer operated at a full-scan mass range *m/z* 40 to 800. For details, see the materials and methods sections in the supplemental material (see Text S1).

### DNA extraction, amplification of 16S rRNA genes, and tag sequencing.

DNA was extracted from approximately 2 g of sediment according to the method of Zhou et al. (65) with the following modifications. For the asphalt sediment, extraction buffer was added and samples were freeze-thawed three times. Samples were sonicated for 10 min and placed in a water bath for 30 min at 65°C. Extraction continued according to the method of Zhou et al., with the modification that the proteinase K concentration was 20 mg ml^−1^ and the incubation temperature for this step was 65°C. Up to 12.5 ng of each DNA extract (ambient, oil, and asphalt sediment) was used for the preparation of 16S rRNA gene amplicon libraries for Illumina sequencing by following the 16S metagenomic sequencing library preparation guide provided by Illumina. Amplicon libraries for both archaea and bacteria were sequenced on an Illumina MiSeq instrument (2× 300-bp paired-end run, v3 chemistry) at CeBiTec (Bielefeld, Germany). The Arch349F and Arch915R primers were used to amplify the archaeal V3 to V5 regions, while for bacteria, the V3 and V4 regions were amplified using Bact 341F and Bact 785R as primers (Table S1). From the retrieved sequences, primer sequences and the remaining adapters were clipped using Cutadapt (66) (v1.9.1), with 0.16 as the maximum allowed error rate (–e) and no indels allowed. For archaea, clipped reads were merged using PEAR (67) (v0.9.6), with 10 bp set as the minimum overlap and 400 and 570 bp set as the minimum and maximum assembly lengths, respectively. Afterwards, merged reads were trimmed using Trimmomatic (68) (v0.35), with 6:12 as the sliding window and 450 bp as the minimum length. For bacteria, clipped reads were first trimmed (sliding window, 4:15; minimum length, 100) and then merged (minimum overlap, 10 bp; minimum length, 350 bp; maximum length, 500 bp) using similar software. Both bacterial and archaeal sequences were then dereplicated and were clustered into operational taxonomic units (OTUs) using Swarm (69) (v2.2.2) with the following parameters: –b 3, –d 1, and –f. Then, OTUs were classified against sequences in the SSURef_NR99_123 SILVA database using SINA aligner (70) (v1.2.11). The classified OTUs were analyzed using the R software.

### CARD-FISH analysis.

Samples for catalyzed reported deposition-fluorescence *in situ* hybridization (CARD-FISH) analysis were fixed on board for 2 h in 2% formaldehyde at 23°C, washed, and stored in phosphate-buffered saline (PBS; pH 7.4)-ethanol (1:1 [vol/vol]) at –20°C. Aliquots were sonicated (30 s, 20% power, 20% cycle, Sonopuls HD70; Bandelin) and filtered on GTTP polycarbonate filters (0.2-μm pore size; Millipore, Darmstadt, Germany). Per filter, 1.3 μl of sediment was used, and several dilution steps allowed an even distribution. The CARD-FISH reaction was performed as described in reference 71, with the following modifications. Cells were permeabilized with a lysozyme solution (PBS [pH 7.4], 0.005 M EDTA [pH 8.0], 0.02 M Tris-HCl [pH 8.0], 10 mg ml^−1^ lysozyme; Sigma-Aldrich) at 37°C for 60 min and either achromopeptidase solution (0.01 M NaCl, 0.01 M Tris-HCl [pH 8.0], 20 μg ml^−1^ achromopeptidase) at 37°C for 30 min or proteinase K solution (PBS [pH 7.4], 0.005 M EDTA [pH 8.0], 0.02 M Tris-HCl [pH 8.0], 15 μg/ml proteinase K; Merck, Darmstadt, Germany) at room temperature for 5 min; endogenous peroxidases were inactivated by incubation in a solution of 0.15% H_2_O_2_ in methanol for 30 min at room temperature. The 16S rRNA was targeted with the specific oligonucleotide probes EUB-338 I-III (72, 73), ARCH-915 (74), ANME-1-350 (10), GOM-ARCI-660, DC06-735, DC06-660, and SYNA-666 (Table S1). The probes EUB-338, ARCH-915, and GOM-ARCI-660 were applied in the hybridization buffer at 35% formamide, the probe ANME-1-350 was applied at 40% formamide, the probe DC06-735 was applied at 10% formamide, and the probes DC06-660 and SYNA-666 were applied at 25% formamide. The probes GOM-ARCI-660, DC06-735, DC06-660, and SYNA-666 were designed in this project by using the probe design tool in the ARB software package to specifically detect “*Ca.* Argoarchaeum” (GoM-Arc1), “*Ca.* Methanoliparia,” and “*Ca.* Syntrophoarchaeum” (Table S1). The probes have at least one mismatch to nontarget groups in the SILVA_132_SSURef_NR99 database. The stringency of the probes was experimentally tested with the Butane50 culture (26) with the probe SYNA-666 and on environmental samples with the probes GOM-ArcI-660, DC06-735, and DC06-660 using 10 to 50% formamide. For the probe SYNA-666, three helper probes (h1SYNA-666 to h3SYNA-666) were applied to increase the performance of the probe together with an unlabeled competitor (c1SYNA-666) targeting marine benthic group E to avoid unspecific binding of the probe. Probe GOM-ARCI-660 was used with three competitors (c1-GOM-ARCI-660 to c3-GOM-ARCI-660). The probes DC06-735 and D-C06-660 were applied without helpers or competitors. The number code of the designed probes refers to the first position of their binding site on the 16S rRNA sequence, with Escherichia coli as the reference. All probes were purchased from biomers.net (Ulm, Germany). Probes were mixed with their corresponding helper and competitor oligonucleotides in the hybridization solution before filter pieces were immersed with a ratio 1:1 (helper/competitor:probe). For double hybridization, the peroxidases from the first hybridization were inactivated by repeating the inactivation step described above. The order of probes used in the double hybridization was independent of the applied formamide concentration. Double hybridization was used to confirm specific binding of the newly designed DC06 probes in oil droplets by using both DC06 probes (Fig. 2F). Double staining with the specific D-C06 probes and the general archaeal probe Arch915 resulted in weak signals for the general probe Arch915, likely due to a single mismatch of the Arch915 probe against the 16S rRNA gene sequences of “*Ca.* Methanoliparia.” The fluorochromes Alexa Fluor 488 and Alexa Fluor 594 were used. Filters were counterstained with DAPI (4′,6′-diamino-2-phenylindole) and analyzed by epifluorescence microscopy (Axiophot II imaging; Zeiss, Germany). Selected filters were analyzed by confocal laser scanning microscopy (model LSM 780; Zeiss, Germany) including the Airyscan technology. Estimated cell abundances were determined by counting signals in 20 grids. To estimate cell abundances in aggregates, spherical aggregate shapes were assumed, with an average diameter in the two-dimensional view and with the understanding that the aggregate contains coccoid cells with a cell size of 1 μm in diameter.

### Metagenomic library preparation, DNA sequencing, and data analysis.

Previously extracted DNA was also used for metagenomics sequencing. For the oil site, DNA from the 9- to 10-cm-depth sediment sample was used to generate 2 PCR-free DNA shotgun libraries with an insert size of 350 bp (Table S2). Additionally, DNA was extracted again from the 9- to 10-cm-depth sediment sample (Table 1, sample 19351-14) as described before and used to generate 7 PCR-free DNA shotgun libraries with insert sizes between 400 and 750 bp. Libraries with the same insert size and sequenced on the same flow cell were merged, resulting in a final number of 6 libraries (Table S2). DNA input amounts were 1 μg for libraries with insert sizes of up to 450 bp and 2 μg for libraries with insert sizes larger than 450 bp. Library preparation was done by following the Illumina TruSeq DNA PCR-free sample preparation guide. The libraries were sequenced on MiSeq (2 × 300 PE [paired end] run, v3 chemistry) and HiSeq 1500 (2 × 250 PE run, rapid v2 SBS [sequencing by synthesis] chemistry) instruments. For the asphalt sediment, one DNA shotgun library with an insert size between 550 and 600 bp was generated from 600 ng DNA from the 135-cm depth, by following the Illumina TruSeq Nano DNA library prep guide. The library was sequenced on a MiSeq instrument (2 × 300 PE run, v3 chemistry). All Illumina sequencing was done at the CeBiTec (Bielefeld, Germany). After being sequenced, primers and adapters were removed from all libraries and quality trimmed using BBDuk from the BBTools package (75), with a minimal *Q* value of 20 and a minimal length of 50 bp. The 16S rRNA gene compositions of all the libraries were estimated using the software phyloFlash v.2.0 (76), with SILVA_128_SSURef_NR99 as the reference database.

### Metagenomic analysis.

The 6 DNA shotgun library sequence data sets from the oil site were used to produce an initial assembly using SPAdes v.3.9.0 (77) with default parameters. Afterwards, the initial assembly was binned using Metawatt 3.5.3 (78) based on tetranucleotide identity, coverage mapping, and coherence of the taxonomic affiliation of the predicted bin proteins. A bin with a 16S rRNA gene affiliated with the “*Ca.* Methanoliparia” clade was extracted. In order to improve the bin quality, targeted reassembly was performed, which consisted of mapping of the reads to the contigs and reassembly of the mapped reads. The reads from the six oil site metagenomic libraries were mapped with 99% identity to the contigs of the extracted bin of each assembly round using BBMap from the BBTools package. Afterwards, the mapped reads were assembled with SPAdes with the flag –careful, and the assembly was binned using Metawatt, discarding contigs below 1,750 bp. Finally, the completeness and contamination of the bin were evaluated using CheckM (79) with the *Euryarchaeota* marker gene set. To improve genome quality, 16 reassembly rounds that successively increased estimated completeness from 59.6% to 75% and slightly decreased contamination from 2.9% to 1.5% were carried out. The sequence data set obtained from the asphalt sediment library was used to produce an initial assembly using SPAdes. As with the oil site, the assembly was binned using Metawatt, and a bin with a 16S rRNA gene affiliated with the “*Ca.* Methanoliparia” clade was extracted. As described before, seven rounds of targeted reassembly were carried out to improve the quality of the bin, resulting in an increase of the completeness from 88% to 92% and an increase of the *N*_50_ value from 2,200 bp to 8,642 bp. Both final bins were used afterwards as metagenome-assembled genomes (MAGs). Genes were predicted and automatically annotated using Prokka (80). Annotation was manually curated using RAST (81), and Pfam and TIGR profiles were identified through HMMER searches (hmmer.org).

### Single-cell sequencing of “*Ca.* Syntrophoarchaeum.”

Anoxic aliquots of sediment samples from the 9- to 10-cm depth of the oil site were shipped at 5°C to the Bigelow Laboratory Single Cell Genomics Center (SCGC; https://scgc.bigelow.org). There, single cells were separated, sorted, and lysed. Afterwards, multiple-displacement amplification was performed and cells containing “*Ca.* Syntrophoarchaeum” were identified by 16S rRNA gene tag sequencing and subsequent genome sequencing (NextSeq 500) as previously described (82). Reads were quality controlled and assembled using SPAdes. The single amplified genome (SAG) was screened for contamination and completeness using CheckM. Gene prediction and annotation were performed as described above for the MAGs of “*Ca.* Methanoliparia.” For more details, see the materials and methods section in the supplemental material (Text S1).

### Phylogenetic analyses of target organisms.

Seven hundred fifty-two full-length 16S rRNA gene sequences from the *Euryarchaeota* obtained from the SILVA_132_SSURef_NR99 database were used for phylogenetic analysis, together with the three 16S rRNA genes of the “*Ca.* Methanoliparia” and “*Ca.* Syntrophoarchaeum” bins obtained in this study. Ten phylogenetic trees were calculated with the ARB software (83) using the maximum-likelihood algorithm RAxML with GTRGAMMA as the model and a 50% similarity filter. One thousand bootstrap analyses were performed to calculate branch support values. The tree with the best likelihood score was selected. For the “*Ca.* Methanoliparia” clade tree, short sequences were added to the calculated tree using the “Quick Add” option from the ARB package, with the default filter for archaea excluding fast-evolving positions and reducing the filter to the 16S rRNA gene boundaries with the terminus filter. To calculate the phylogenetic affiliations of methyl-coenzyme M reductase subunit alpha (*mcrA*), methylcobalamin:coenzyme M methyltransferase (*mtaA/mtbA*) and methyl-H_4_MPT:coenzyme M methyltransferase subunit H (*mtrH*), the corresponding protein sequences present in the bins of “*Ca.* Methanoliparia” and “*Ca.* Syntrophoarchaeum” were aligned to protein sequences of the corresponding genes obtained from the NCBI database (332 McrA sequences, 527 MtaA/MtbA sequences, and 169 MtrH sequences). Sequences were aligned using the software MUSCLE (84) v3.7, followed by manual refinement. Afterwards, a masking filter over the common regions of every alignment was calculated using Zorro (85). Trees were calculated using the maximum-likelihood algorithm RAxML (86), v.8.2.11, and the masking filter with the automatic protein model assignment algorithm PROTGAMAAUTO, which always selected LG as the amino acid substitution model. Bootstrap analyses were performed according to the convergence criterion of RAxML, with 99 iterations performed for McrA, 149 for Mta, and 100 for MtrH. The resulting trees were displayed with the iTOL Web server (87).

### Data availability.

Sequence data were deposited in the European Nucleotide Archive (ENA) (88) using the data brokerage service of the German Federation for Biological Data (GFBio) (89), in compliance with the minimal information about any (X) sequence (MIxS) standard (90). All reads and genomic information, including metagenomic, single-cell, and 16S rRNA gene tag reads, have been stored under the study number PRJEB32776. Draft genomes of the described organisms can be found under the same study number with the accession numbers GCA_902158735 (Methanoliparia_GoM_oil), GCA_902158745 (Methanoliparia_GoM_asphalt), and GCA_902158755 (Syntropho_SAG).
