# Supplementary material for: Anaerobic Degradation of Non-Methane Alkanes by “Candidatus Methanoliparia” in Hydrocarbon Seeps of the Gulf of Mexico
Source: mBio. 2019 Aug 20;10(4):e01814-19. doi: 10.1128/mBio.01814-19 (PMC6703427; doi:10.1128/mBio.01814-19)
Supplement: TEXT S1 [file mBio.01814-19-s0001.docx]

**Supplementary Text**

***Supplementary Material and Methods***

**Measurements of short-chain alkanes.** For analysis of short-chain alkanes (C_1_ to *n-*C_6_) in push core GeoB19351-14 (oily sediments), a modified headspace technique was used (1). Briefly, 3 ml of sediment were taken immediately after sample recovery from the selected depth intervals using tip-cut syringes, and transferred to 20 ml crimp vials filled with 5 ml sodium hydroxide solution. Vials were closed with butyl rubber septa. Concentrations of short-chain alkanes in the headspace gas were determined by gas chromatography (GC) on an Agilent 6890N gas chromatograph (injector temperature: 180°C) with a capillary column (OPTIMA-5, Macherey-Nagel, 50m × 0.32mm, 5µm film thickness; carrier gas: helium, const. pressure 1.4 bar) coupled to a flame ionization detector (T: 250°C) and referred to volume of bulk sediment (2). Calibrations and performance checks of the analytical system were conducted daily using commercial pure gas standards and gas mixtures (Air Liquide, Germany) of known compositions. The analytical error estimated by multiple injections of gas standards was <2.0% for each signal. Stable carbon isotope ratios (^13^C/^12^C) of CH_4_ in two headspace samples from push core GeoB19351-14 were determined by GC-isotope ratio mass spectrometry on a Trace GC Ultra (injector temperature: 180°C) connected via a GC IsoLink (combustion oven temperature: 1,030°C) and a ConFlo IV interface to a MAT 253 isotope mass spectrometer (all components Thermo Fisher Scientific). For compound separation, a CARBOXEN-1006 PLOT (Supelco Inc., 30m × 0.32µm) capillary column was used. Helium was the carrier gas and the flow was 1.0 ml min^-1^. Reported ^13^C/^12^C values are arithmetic means of duplicate measurements at least in the δ-notation relative to Vienna-Pee Dee Belemnite (V-PDB) and SMOW standard, respectively. Reproducibility was checked daily using commercial pure methane standards (Isometric Instruments, Canada; Air Liquide GmbH, Germany) of known concentrations and stable carbon isotopic compositions. Standard deviations of triplicate stable isotope measurements were δ^13^C-CH_4_<0.5‰.

**Rates of methane oxidation and sulfate reduction.** To determine rates of methane oxidation and sulfate reduction, specific depth horizons of the push cores from the oil sites were subsampled into small gas tight cylinders closed with septum and plungers. Radiolabelled (^14^C)-methane and (^35^S)-sulfate were added in three replicates and killed controls, respectively, and samples were incubated at 4°C for 24 hours. To stop the ^14^C-incubations, samples were transferred to gas-tight bottles filled with sodium hydroxide solution (2.5%). To determine rates of methane oxidation, methane concentrations were measured in the samples by gas chromatography (Focus GC Thermo). Tracer content in methane and dissolved inorganic carbon were determined by methods described in Treude et al. (2003) (3). To stop the ^35^S-incubations, samples were transferred to zinc acetate (2%) solution. Tracer content in sulfate was determined from the supernatant. The product of the reaction, reduced sulfur, was released from the sample and collected using the cold chromium approach (4). Radioactivity in the samples was measured by scintillation counting (Scintillation cocktail Ultima Gold; scintillation counter 2900TR LSA; Packard). Concentrations of sulfate (porewater samples from replicate cores) were determined using non-suppressed ion chromatography (Metrohm 760c). Rates were calculated as described previously(5).

**Patterns of long-chain hydrocarbons in oily sediments and asphalt flow.** The organic fraction of the oily sediments and the asphalt flows was extracted with dichloromethane (DCM) in an ultrasonic bath for 10 minutes. Asphaltenes were precipitated in three steps by addition of cold *n*-hexane. The *n*-hexane fraction was subsequently subjected to silica gel column chromatography and saturated hydrocarbons were eluted with *n*-hexane. The saturated fraction was analysed on a ThermoFinnigan Trace GC equipped with a 30 m RTX-5MS fused silica column (0.25 mm, 0.25 µm) coupled to a ThermoFinnigan TraceMS. The MS was operated in electron impact mode at 70 eV with a full scan mass range of 40-800 *m/z*. The initial oven temperature was held at 60°C for 2 minutes and subsequently heated to 325°C at a rate of 4°C per min and held at 325°C for 20 minutes. The carrier gas was helium with a constant flow of 1.0 ml min^-1^.

**Single cell sequencing of *Ca.* Syntrophoarchaeum.** Anoxic aliquots of sediment samples from the 9-10 cm depth of the oil site were shipped at 5°C to the Bigelow Laboratory Single Cell Genomics Center (SCGC; https://scgc.bigelow.org). There, cells were separated from the sediment after dilution by centrifugation. Then, by high-speed fluorescence-activated and droplet-based cell sorting (FACS) single cells were sorted into a 384-well plate, where cell were lysed by five freeze-thaw cycles and KOH treatment. Multiple displacement amplification of the single cell genomic DNA was performed followed by phylogenetic identification of *Ca.* Syntrophoarchaeum cells and subsequent genome sequencing as previously described (6). Phylogenetic identification was performed by 16S rRNA gene tag sequencing. Only one cell affiliated with the *Ca.* Syntrophoarchaeum clade. For genome sequencing, libraries were constructed with Nextera XT (Illumina) and then sequenced with NextSeq 500. Afterwards, reads were quality controlled using Trimmomatic (7) v0.32 (‘-phred33 LEADING:0 TRAILING:5 SLIDINGWINDOW:4:15 MINLEN:36’) and searched for human contamination. The quality-controlled reads were assembled using SPAdes with the parameters --careful --sc --phred-offset 33 after normalizing the read kmer coverage using kmernorm 1.05 (<https://sourceforge.net/projects/kmernorm/>) with the flags -k 21 -t 30 -c 3. After assembly, contigs below 2200 bp were discarded. The final bin was screened for contamination and completeness using CheckM. Afterwards, it was considered as a single-amplified genome (SAG). Gene prediction and annotation was performed as described above for the MAGs of *Ca.* Methanoliparia. Cells affiliated to *Ca.* Methanoliparia could not be sorted, likely because these archaea are associated to the oil droplets, which were not available to different procedures of cell sorting.

***Supplementary Results and Discussion***

**Hydrocarbon degradation in oil- and asphalt-rich sediments**

All samples of this study were obtained from marine sediments and asphalt deposits of the Campeche hydrocarbon province in the Gulf of Mexico (Table 1). The main sample for this study, termed “oily sediment” (GeoB19351-14; Figure S1C) was obtained by push coring in the direct vicinity of the Chapopote asphalt volcano (8, 9). Emerging oily gas bubbles and partly oil-coated gas hydrate outcrops showed the hydrocarbon-rich nature of this habitat, and abundant sulfide-dependent chemosynthetic seep fauna suggested intense hydrocarbon-dependent sulfate reduction in the underlying sediment. The sample contained large amounts of petroleum hydrocarbons. Short-chain alkanes such as methane (>2500 µmol per liter sediment), ethane (around 360 μM), propane and *i*-butane (both around 50 μM) were particularly abundant in the deeper sediment horizons at 6 and 8 cm below seafloor (cmbsf; Figure S1A). The anaerobic oxidation of methane in the sediments created a sulfate-methane transition zone at approximately 3-6 cmbsf. Rates of sulfate reduction measured in ^35^S-sulfate incubations exceeded rates of methane oxidation measured by^14^C-methane tracer incubations by up to 4-fold, indicating that a substantial fraction of the sulfate reduction was coupled to the oxidation of non-methane hydrocarbons, as observed before for other Gulf of Mexico hydrocarbon seeps (10, 11). Long-chain alkanes were largely depleted throughout the core (Figure S1B). Intense biodegradation in this core was also inferred from the pronounced *iso*-butane over *n*-butane prevalence in the sample from 6 cmbsf (*i*-C_4_/*n*-C_4_ ~ 17) and the virtual absence of *n*-butane in all other samples (c.f (12)). Furthermore, methane in the studied core was slightly depleted in ^13^C (δ^13^C-CH_4_ –55.4 to –55.5‰; Figure S1A) compared to methane in the surrounding methane seeps (-46.5‰, (13)), suggesting biogenic methane production, likely coupled to the degradation of long-chain alkanes. Indeed, previous studies in similar sediments of Chapopote revealed methane δ^13^C values as negative as −65‰, indicating that biogenic methanogenesis can be even more pronounced at this site (14, 15).

The second sample, “asphalt flow” sample (GeoB19351-14), was collected from the main asphalt field of the Chapopote asphalt volcano. These asphalts lacked free water and only traces of DNA could be extracted from those samples. Furthermore, they showed no sign of biodegradation, as a full mix of alkanes was still present. Accordingly, we conclude that these freshly deposited asphalts did not yet undergo substantial microbial alteration and we consider them as a reference for the original hydrocarbon composition. In contrast to the oily sediments, the asphalt flows still contained a mix of alkanes (C_12-38_) (Figure S1B). The third sample, “ambient sediments” sample (GeoB19351-5), was retrieved from nearby sediment with no exposure to oil or other hydrocarbons (Figure S1D). This sample was light-grey, non-sulfidic and contained only trace amounts of methane and no oil. The sediment coloring suggested that oxygen penetrated several centimeters deep and sulfate was not consumed. The fourth sample, “asphalt sediment” sample GeoB19331-1, was retrieved using a gravity core from the Mictlan asphalt volcano situated northeast of Chapopote (15, 16). The upper part contained normal pelagic sediment. In the bottom, at 135 cm sediment depth, solid asphalt pieces were retrieved to study their microbial communities.

**Bacterial and archaeal proportional abundances in the sediments**

To characterize the microbial community in three of the sites (oily, ambient and asphalt sediments), 16S rRNA gene amplicon libraries for bacteria and archaea were obtained and sequenced. The bacterial library from the ambient sediments contained mostly *Epsilonproteobacteria* (24-33%) and *Gammaproteobacteria* (10-17%), while the one from the oily sediments contained mostly *Deltaproteobacteria* (23-32%) affiliated with *Desulfobacterales* (13-17%), *Desulfarculales* (6-7%) and *Syntrophobacterales* (3-5%) (Supplementary Dataset_1A)*.* Some of their members are considered to be important anaerobic alkane degraders (17-20). A large portion of these sequences clustered with the Seep-SRB 1 (3-7%) and Seep-SRB 2 (3-5%) clades (Figure 1A), which include the most abundant sulfate-reducing partner bacteria of ANME archaea in AOM (17, 21). These partner bacteria are autotrophs and do not contain pathways for hydrocarbon degradation (22, 23). The asphalt sediments contained only small proportions of these Seep-SRB clades (<2%). Instead they were dominated by *Atribacteria* (53-56%), which are heterotrophic anaerobes that have been repeatedly found in hydrocarbon-rich environments (24).

The archaeal 16S rRNA gene libraries of the ambient sediments were dominated by *Thaumarchaeota* (24-47%) and *Woesearchaeota* (15-65%) with a small proportion of *Thermoplasmata* (9-32%) (Supplementary Dataset_1B). ANME archaea could not be detected in the ambient sediments. By contrast, the oily sediments showed high sequence proportions of the euryarchaeotal groups ANME-1 (17-33%) and *Ca.* Methanoliparia (8-23%) plus considerable proportions of sequences affiliated with the Marine Benthic Group B (*Thaumarchaeota*; 12-21%), ANME-2c (2-7%), *Ca.* Argoarchaeum (GoM-Arc1; 3-6%) and Marine Group II (14-23%), the latter belonging to the *Thermoplasmatales* (Figure 1B). Furthermore, we found low abundances of *Ca.* Syntrophoarchaeum (<1%). The asphalt sediments were dominated by ANME-1 sequences (77-92%) with considerable proportions of *Ca.* Methanoliparia (6-7%).

The high relative sequence abundance of ANME archaea at the oily and asphalt sites underlines AOM as one of the main sulfate sinks in these sediments. It was speculated that some of the members of the ANME-1 clade may also thrive as methanogens or degraders of non-methane hydrocarbons as suggested by geochemical and molecular profiling of sediments and metagenomics (25, 26, 27). However, we measured substantial AOM rates in the sediments and there were relatively small proportions of other ANME archaea (ANME-2c: 2-7%), pointing to the role of ANME-1 as methane oxidizers in these sediments. The presence of alkane-degrading archaea, like *Ca.* Syntrophoarchaeum and *Ca.* Argoarchaeum, remarks the environmental role of these organisms in anaerobic short-chain alkane degradation. The high proportional abundance of *Ca.* Methanoliparia in these sediments supports a potential role of this clade in the degradation of oil.

**Genomic information of the SAG affiliated to Ca. Syntrophoarchaeum**

A single amplified genome (SAG) affiliated to *Ca.* Syntrophoarchaeum was retrieved from the oily sediments from the 9-10 cm depth based on single cell sorting followed by sequencing. The phylogenetic affiliation of the SAG was based on the 16S rRNA gene amplification. The retrieved SAG (Syntropho_SAG) was 42.7% complete and had no contamination (Table S3). Based on its average nucleotide identity (74-84%, Table S3) and its 16S rRNA gene identity (96-97%) compared to the described species of the genus *Ca.* Syntrophoarchaeum, this SAG likely represents a new species within the genus.

The 16S rRNA gene of the Syntropho_SAG was affiliated to the *Ca.* Syntrophoarchaeum clade in the 16S rRNA gene phylogeny (Figure 3A-B). The clade was located within the *Methanomicrobia* and included several sequences from different environments like the Gulf of Mexico and the Guaymas Basin. *Ca.* Syntrophoarchaeum should be a separate order based on the identity values to the sister groups ANME-1 and *Methanosarcinales* of 81-86%. Recent phylogenetic analyses of single-copy marker genes support these results, placing *Ca.* Syntrophoarchaeum next to ANME-1 and close to the *Methanomicrobia* (28)*.*

The genomic metabolic potential is largely similar to previously published MAGs of Ca. Syntrophoarchaeum (29), although not all genes necessary for alkane degradation are found (Figure S3), which is likely due to its low completeness. For instance, operons with *mcrABG* are missing, yet other genes essential for the alkane-based catabolism were present including *mcrC, metFV*, heterodisulfide reductases complexes, divergent cobalamin methyltransferases (Figure 6B) and genes encoding the last steps of the reverse methanogenesis like formylmethanofuran dehydrogenase (*fmd*) and formylmethanofuran-tetrahydromethanopterin formyltransferase (*ftr*). Moreover, the Syntropho_SAG encodes for a complete fatty acid oxidation including several copies encoding some of the steps, what was proposed as a sign of potential degradation of different multi-carbon compounds (29).

**Supplementary References**

1. Pape T, Geprägs P, Hammerschmidt S, Wintersteller P, Wei J, Fleischmann T, Bohrmann G, Kopf AJ. 2014. Hydrocarbon seepage and its sources at mud volcanoes of the Kumano forearc basin, Nankai Trough subduction zone. Geochemistry, Geophysics, Geosystems 15:2180-2194.

2. Pape T, Bahr A, Rethemeyer J, Kessler JD, Sahling H, Hinrichs K-U, Klapp SA, Reeburgh WS, Bohrmann G. 2010. Molecular and isotopic partitioning of low-molecular-weight hydrocarbons during migration and gas hydrate precipitation in deposits of a high-flux seepage site. Chemical Geology 269:350-363.

3. Treude T, Boetius A, Knittel K, Wallmann K, Jorgensen BB. 2003. Anaerobic oxidation of methane above gas hydrates at Hydrate Ridge, NE Pacific Ocean. Marine Ecology Progress Series 264:1-14.

4. Kallmeyer J, Ferdelman TG, Weber A, Fossing H, Jørgensen BB. 2004. A cold chromium distillation procedure for radiolabeled sulfide applied to sulfate reduction measurements. Limnology and Oceanography: Methods 2:171-180.

5. Holler T, Widdel F, Knittel K, Amann R, Kellermann MY, Hinrichs K-U, Teske A, Boetius A, Wegener G. 2011. Thermophilic anaerobic oxidation of methane by marine microbial consortia. The ISME journal 5:1946.

6. Stepanauskas R, Fergusson EA, Brown J, Poulton NJ, Tupper B, Labonté JM, Becraft ED, Brown JM, Pachiadaki MG, Povilaitis T, Thompson BP, Mascena CJ, Bellows WK, Lubys A. 2017. Improved genome recovery and integrated cell-size analyses of individual uncultured microbial cells and viral particles. Nature Communications 8:84.

7. Bolger AM, Lohse M, Usadel B. 2014. Trimmomatic: a flexible trimmer for Illumina sequence data. Bioinformatics 30:2114-2120.

8. MacDonald IR, Bohrmann G, Escobar E, Abegg F, Blanchon P, Blinova V, Brückmann W, Drews M, Eisenhauer A, Han X, Heeschen K, Meier F, Mortera C, Naehr T, Orcutt B, Bernard B, Brooks J, de Faragó M. 2004. Asphalt Volcanism and Chemosynthetic Life in the Campeche Knolls, Gulf of Mexico. Science 304:999-1002.

9. Marcon Y, Sahling H, MacDonald IR, Wintersteller P, dos Santos Ferreira C, Bohrmann G. 2018. Slow volcanoes: The intriguing similarities between marine asphalt and basalt lavas. Oceanography 31.

10. Joye SB, Boetius A, Orcutt BN, Montoya JP, Schulz HN, Erickson MJ, Lugo SK. 2004. The anaerobic oxidation of methane and sulfate reduction in sediments from Gulf of Mexico cold seeps. Chemical Geology 205:219-238.

11. Orcutt BN, Joye SB, Kleindienst S, Knittel K, Ramette A, Reitz A, Samarkin V, Treude T, Boetius A. 2010. Impact of natural oil and higher hydrocarbons on microbial diversity, distribution, and activity in Gulf of Mexico cold-seep sediments. Deep Sea Research Part II: Topical Studies in Oceanography 57:2008-2021.

12. James A, Burns B. 1984. Microbial alteration of subsurface natural gas accumulations. AAPG Bulletin 68:957-960.

13. Sahling H, Blum MR, Borowski C, Escobar-Briones E, Gaytán-Caballero A, Hsu C-W, Loher M, MacDonald I, Marcon Y, Pape T. 2016. Seafloor observations at Campeche Knolls, southern Gulf of Mexico: coexistence of asphalt deposits, oil seepage, and gas venting. Biogeosciences 13:4491-4512.

14. Schubotz F, Lipp JS, Elvert M, Kasten S, Mollar XP, Zabel M, Bohrmann G, Hinrichs K-U. 2011. Petroleum degradation and associated microbial signatures at the Chapopote asphalt volcano, Southern Gulf of Mexico. Geochimica et Cosmochimica Acta 75:4377-4398.

15. Sahling H, Borowski C, Escobar-Briones E, Gaytán-Caballero A, Hsu C-W, Loher M, MacDonald I, Marcon Y, Pape T, Römer M. 2016. Massive asphalt deposits, oil seepage, and gas venting support abundant chemosynthetic communities at the Campeche Knolls, southern Gulf of Mexico. Biogeosciences 13:4491-4512.

16. Sahling H, Ohling G. 2017. R/V Meteor Cruise Report M114: Natural Hydrocarbon Seepage in the Southern Gulf of Mexico: Kingston-Kingston, 12 February-28 March 2015: Cruise Sponsored by Deutsche Forschungsgemeinschaft (DFG). MARUM-Zentrum für Marine Umweltwissenschaften, Fachbereich Geowissenschaften, Universität Bremen.

17. Kleindienst S, Ramette A, Amann R, Knittel K. 2012. Distribution and in situ abundance of sulfate‐reducing bacteria in diverse marine hydrocarbon seep sediments. Environmental microbiology 14:2689-2710.

18. Kleindienst S, Herbst F-A, Stagars M, von Netzer F, von Bergen M, Seifert J, Peplies J, Amann R, Musat F, Lueders T, Knittel K. 2014. Diverse sulfate-reducing bacteria of the Desulfosarcina/Desulfococcus clade are the key alkane degraders at marine seeps. The ISME journal 8:2029.

19. Knittel K, Boetius A, Lemke A, Eilers H, Lochte K, Pfannkuche O, Linke P, Amann R. 2003. Activity, Distribution, and Diversity of Sulfate Reducers and Other Bacteria in Sediments above Gas Hydrate (Cascadia Margin, Oregon). Geomicrobiology Journal 20:269-294.

20. Stagars MH, Ruff SE, Amann R, Knittel K. 2016. High Diversity of Anaerobic Alkane-Degrading Microbial Communities in Marine Seep Sediments Based on (1-methylalkyl)succinate Synthase Genes. Frontiers in microbiology 6.

21. Schreiber L, Holler T, Knittel K, Meyerdierks A, Amann R. 2010. Identification of the dominant sulfate‐reducing bacterial partner of anaerobic methanotrophs of the ANME‐2 clade. Environmental microbiology 12:2327-2340.

22. Krukenberg V, Riedel D, Gruber-Vodicka HR, Buttigieg PL, Tegetmeyer HE, Boetius A, Wegener G. 2018. Gene expression and ultrastructure of meso- and thermophilic methanotrophic consortia. Environmental microbiology 20:1651-1666.

23. Petro C, Jochum LM, Schreiber L, Marshall IPG, Schramm A, Kjeldsen KU. 2019. Single-cell amplified genomes of two uncultivated members of the deltaproteobacterial SEEP-SRB1 clade, isolated from marine sediment. Marine Genomics doi:https://doi.org/10.1016/j.margen.2019.01.004.

24. Nobu MK, Dodsworth JA, Murugapiran SK, Rinke C, Gies EA, Webster G, Schwientek P, Kille P, Parkes RJ, Sass H, Jørgensen BB, Weightman AJ, Liu W-T, Hallam SJ, Tsiamis G, Woyke T, Hedlund BP. 2015. Phylogeny and physiology of candidate phylum ‘Atribacteria’ (OP9/JS1) inferred from cultivation-independent genomics. The ISME journal 10:273.

25. Lloyd KG, Alperin MJ, Teske A. 2011. Environmental evidence for net methane production and oxidation in putative ANaerobic MEthanotrophic (ANME) archaea. Environmental microbiology 13:2548-2564.

26. Dombrowski N, Teske AP, Baker BJ. 2018. Expansive microbial metabolic versatility and biodiversity in dynamic Guaymas Basin hydrothermal sediments. Nature Communications 9:4999.

27. Beulig F, Røy H, McGlynn S, Jørgensen B. 2018. Cryptic CH4 cycling in the sulfate–methane transition of marine sediments apparently mediated by ANME-1 archaea. ISME J 296:10.1038.

28. Adam PS, Borrel G, Brochier-Armanet C, Gribaldo S. 2017. The growing tree of Archaea: new perspectives on their diversity, evolution and ecology. The ISME journal 11:2407.

29. Laso-Pérez R, Wegener G, Knittel K, Widdel F, Harding KJ, Krukenberg V, Meier DV, Richter M, Tegetmeyer HE, Riedel D, Richnow H-H, Adrian L, Reemtsma T, Lechtenfeld OJ, Musat F. 2016. Thermophilic archaea activate butane via alkyl-coenzyme M formation. Nature 539:396.
