# Supplementary figures and images for: Anaerobic Degradation of Non-Methane Alkanes by “Candidatus Methanoliparia” in Hydrocarbon Seeps of the Gulf of Mexico
Source: mBio. 2019 Aug 20;10(4):e01814-19. doi: 10.1128/mBio.01814-19 (PMC6703427; doi:10.1128/mBio.01814-19)

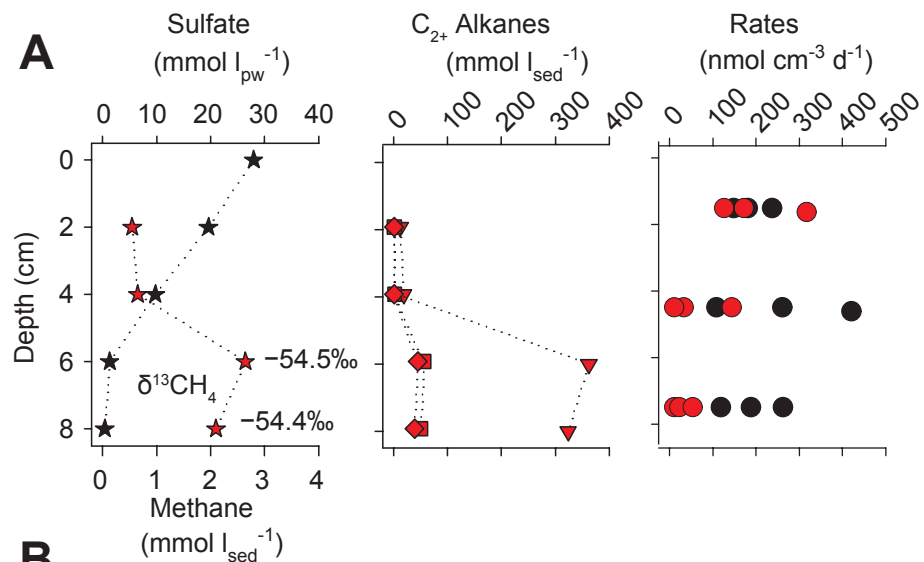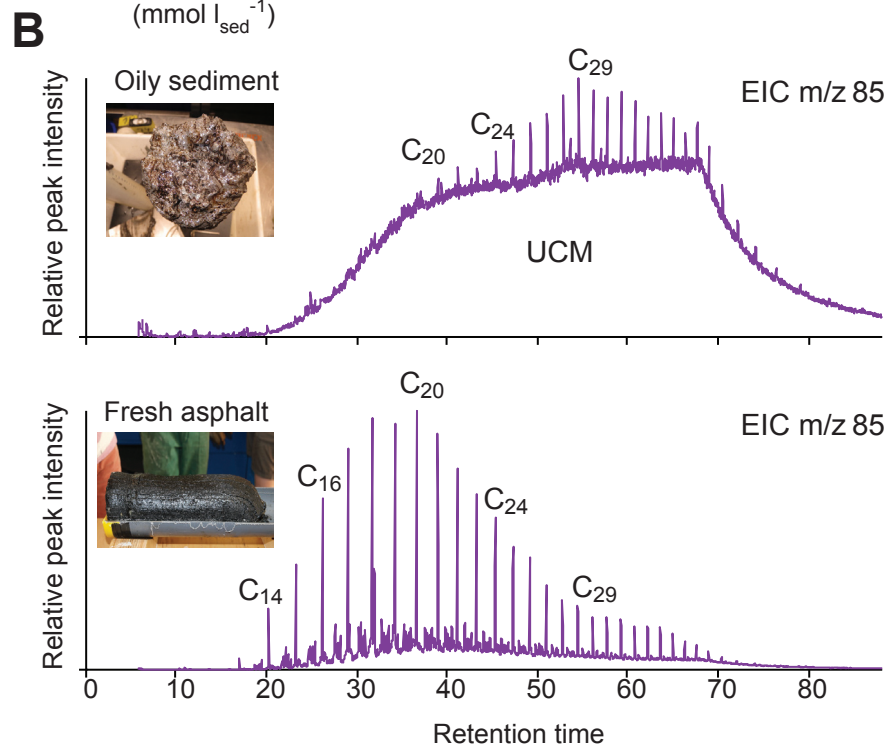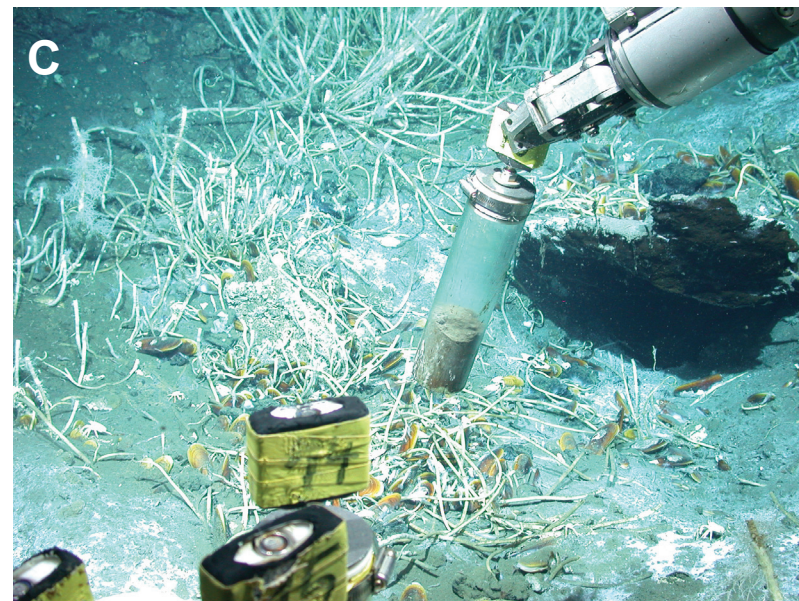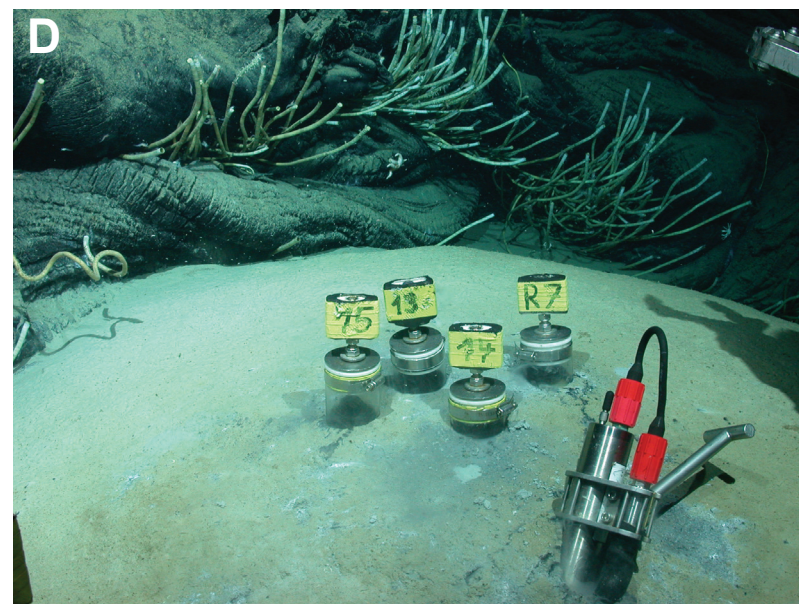

Supplement: FIG S1 [file mBio.01814-19-sf001.pdf]

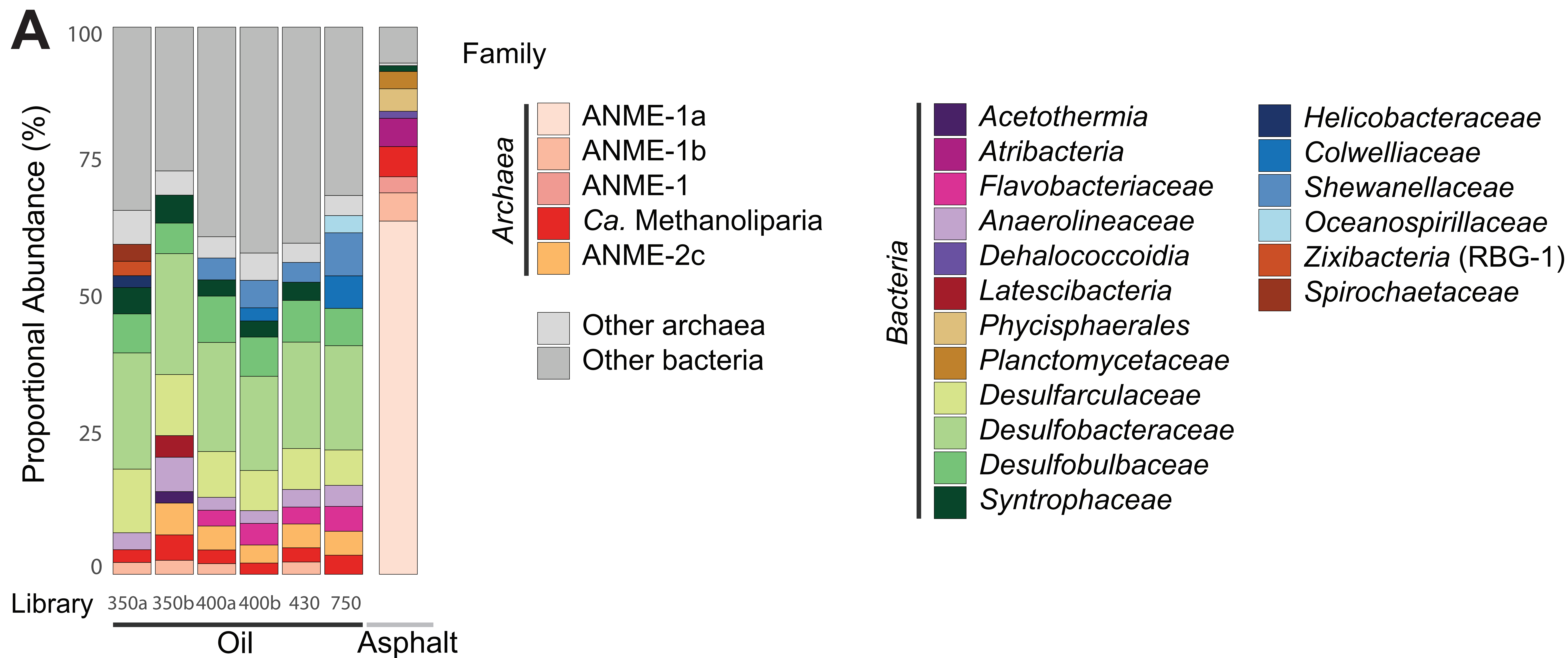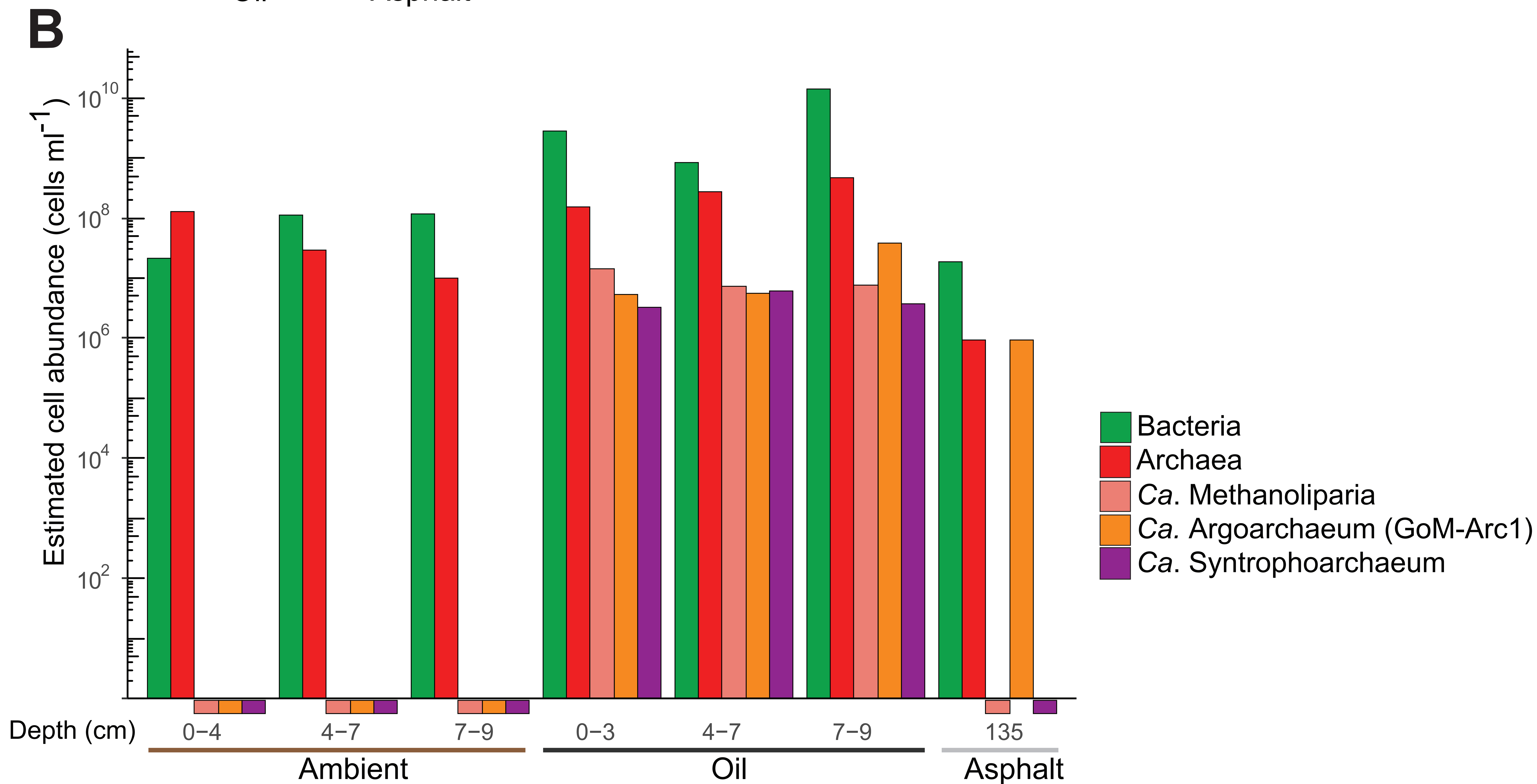

Supplement: FIG S2 [file mBio.01814-19-sf002.pdf]
