# Supplementary material for: Anaerobic Degradation of Non-Methane Alkanes by “Candidatus Methanoliparia” in Hydrocarbon Seeps of the Gulf of Mexico
Source: mBio. 2019 Aug 20;10(4):e01814-19. doi: 10.1128/mBio.01814-19 (PMC6703427; doi:10.1128/mBio.01814-19)
Supplement: TABLE S1 [file mBio.01814-19-st001.docx]

**Table S1.** CARD-FISH probes, competitors and helpers applied in this study and primers used for the PCR sequencing of the 16S rRNA gene amplicon libraries prior to Illumina sequencing. FA% = Formamide concentration.

| **Name** | **Sequence 5’‒>3’** | **Specificity** | **FA%** | **Reference** |
| --- | --- | --- | --- | --- |
| EUB338 I | GCTGCCTCCCGTAGGAGT | Bacteria | 35 | (1) |
| EUB338 II | GCAGCCACCCGTAGGTGT | supplement to EUB338 | 35 | (2) |
| EUB338 III | GCTGCCACCCGTAGGTGT | supplement to EUB338 | 35 | (2) |
| ARCH915 | GTGCTCCCCCGCCAATTCCT | Archaea | 35 | (3) |
| ANME-1-350 | AGTTTTCGCGCCTGATGC | ANME-1 | 40 | (4) |
| GOM-ARCI-660 | AGTACCTCCTACCTCTCCC | most *Ca.* Argoarchaeum (GoM-Arc1); *Methanocellaceae* | 35 | This study |
| c1GOM-ARCI-660 | AGTACCTCCCACCTCTCCC | most ANME-2d (competitor) |  | This study |
| c2GOM-ARCI-660 | AGTACCTCCAACCTCTCCC | *Methanosaetaceae* and *Methanobacteriales* (competitor) |  | This study |
| c3GOM-ARCI-660 | AGTACCTCCGACCTCTCCC | Various archaeal groups (competitor) |  | This study |
| DC06-735 | CGAACCTGTTCTAACTAG | *Ca.* Methanoliparia | 10 | This study |
| DC06-660 | ATACCTCTAGTCTCTCCC | *Ca.* Methanoliparia | 25 | This study |
| SYNA666 | CCTGAAGTACCTCCAACC | *Ca.* Syntrophoarchaeum | 25 | This study |
| c1SYNA666 | CCTGAAGTACCTCAAACC | Marine Benthic Group E (competitor) |  | This study |
| h1SYNA666 | AGACCCGTTCCAGTTGGA | Helper |  | This study |
| h2SYNA666 | AGACCCGTCCCAGTTGGA | Helper |  | This study |
| h3SYNA666 | CCCAGGGATCACAGGATT | Helper |  | This study |
| **ID name** | **Sequence 5’‒>3’** | **Name in probeBase database** | | **Reference** |
| Arch 349F | GYG CAS CAG KCG MGA AW | S-D-Arch-0349-a-S-17 | | (5) |
| Arch915R | GTG CTC CCC CGC CAA TTC CT | S-D-Arch-0911-a-A-20 | | (3) |
| Bact 341F | CCT ACG GGN GGC WGC AG | S-D-Bact-0341-b-S-17 | | (6) |
| Bact 785R | GAC TAC HVG GGT ATC TAA TCC | S-D-Bact-0785-a-A-21 | | (6) |

**References**

1. Amann RI, Binder BJ, Olson RJ, Chisholm SW, Devereux R, Stahl DA. 1990. Combination of 16S rRNA-targeted oligonucleotide probes with flow cytometry for analyzing mixed microbial populations. Applied and environmental microbiology 56:1919-1925.

2. Daims H, Brühl A, Amann R, Schleifer K-H, Wagner M. 1999. The Domain-specific Probe EUB338 is Insufficient for the Detection of all Bacteria: Development and Evaluation of a more Comprehensive Probe Set. Systematic and applied microbiology 22:434-444.

3. Stahl DA, Amann R. 1991. p 205-248. *In* Stackebrandt E, Goodfellow M (ed), Nucleic Acid Techniques in Bacterial Systematics. John Wiley & Sons, Chichester.

4. Boetius A, Ravenschlag K, Schubert CJ, Rickert D, Widdel F, Gieseke A, Amann R, Jørgensen BB, Witte U, Pfannkuche O. 2000. A marine microbial consortium apparently mediating anaerobic oxidation of methane. Nature 407:623.

5. Takai K, Horikoshi K. 2000. Rapid Detection and Quantification of Members of the Archaeal Community by Quantitative PCR Using Fluorogenic Probes. Applied and environmental microbiology 66:5066-5072.

6. Herlemann DPR, Labrenz M, Jürgens K, Bertilsson S, Waniek JJ, Andersson AF. 2011. Transitions in bacterial communities along the 2000 km salinity gradient of the Baltic Sea. The ISME journal 5:1571.
