# Supplementary material for: Anaerobic Degradation of Non-Methane Alkanes by “Candidatus Methanoliparia” in Hydrocarbon Seeps of the Gulf of Mexico
Source: mBio. 2019 Aug 20;10(4):e01814-19. doi: 10.1128/mBio.01814-19 (PMC6703427; doi:10.1128/mBio.01814-19)
Supplement: TABLE S2 [file mBio.01814-19-st002.docx]

**Table S2.** Metagenomic libraries used for the assembly of Methanoliparia_GoM MAGs

| **Library name** | **Sample (GeoB no.)** | **Sequencing platform** | **Read length (bp)** | **Insert size (bp)** | **Read number (PE)** |
| --- | --- | --- | --- | --- | --- |
| TSf_CH14_9-10_350a | Oily sediments (19351-14) | MiSeq | 2×300 | 350 | 2,034,558 |
| TSf_CH14_9-10_350b | Oily sediments (19351-14) | MiSeq | 2×300 | 350 | 1,338,251 |
| TSf_CH14_9-10_400a | Oily sediments (19351-14) | HiSeq | 2×250 | 400 | 41,204,810 |
| TSf_CH14_9-10_400b | Oily sediments (19351-14) | HiSeq | 2×250 | 400 | 59,942,642 |
| TSf_CH14_9-10_430 | Oily sediments (19351-14) | HiSeq | 2×250 | 430 | 19,480,273 |
| TSf_CH14_9-10_750 | Oily sediments (19351-14) | HiSeq | 2×250 | 750 | 2,194,219 |
| GoMasph_12 | Asphalt sediments (19331-1) | MiSeq | 2×300 | 550-600 | 2,354,296 |
