# Supplementary material for: Anaerobic Degradation of Non-Methane Alkanes by “Candidatus Methanoliparia” in Hydrocarbon Seeps of the Gulf of Mexico
Source: mBio. 2019 Aug 20;10(4):e01814-19. doi: 10.1128/mBio.01814-19 (PMC6703427; doi:10.1128/mBio.01814-19)
Supplement: TABLE S3 [file mBio.01814-19-st003.docx]

**Table S3.** Genomic information from the extracted MAGs (Methanoliparia_GoM_oil and Methanoliparia_GoM_asphalt) and the Syntropho_SAG affiliated to *Ca.* Syntrophoarchaeum and pairwise whole genome identity comparison between Methanoliparia_GoM MAGs and the genomes of *Ca.* M. hydrocarbonicum and *Ca.* M. thermophilum; and between the Syntropho_SAG and the genomes of *Ca.* Syntrophoarchaeum butanivorans and *Ca.* Syntrophoarchaeum caldarius. ANI: average nucleotide identity

|  | **Methanoliparia_GoM_oil** | **Methanoliparia_GoM_asphalt** | | | **Syntropho_SAG** | |
| --- | --- | --- | --- | --- | --- | --- |
| Size (bp) | 1703955 | 1753872 | | | 816919 | |
| Scaffolds/Contigs | 218/218 | 270/271 | | | 46/46 | |
| GC content (%) | 42.6 | 42.7 | | | 44.1 | |
| Scaffold N50 (bp) | 10468 | 8642 | | | 33143 | |
| Number of ORFs | 1827 | 1978 | | | 861 | |
| rRNAs | 5S, 16S, 23S | 5S, 16S, 23S | | | 5S, 16S and 23S | |
| tRNAs | 39 | 48 | | | 23 | |
| Completeness (%)^1^ | 75.2 | 92.24 | | | 42.7 | |
| Contamination (%)^1^ | 1.47 | 1.73 | | | 0.0 | |
| Strain heterogeneity (%)^1^ | 66.67 | 20.0 | | | 0.0 | |
|  | | | **ANI**  **(Blast)** | **ANI (MUMmer)** | | **Tetranucleotide frequency** |
| Methanoliparia_GoM_oil/Methanoliparia_GoM_asphalt | | | 93.44 | 94.84 | | 0.9951 |
| Methanoliparia_GoM_asphalt/Methanoliparia_GoM_oil | | | 93.37 | 94.83 | | 0.9951 |
| Methanoliparia_GoM_oil/M.hydrocarbonicum | | | 92.00 | 93.76 | | 0.99428 |
| Methanoliparia_GoM_oil/M,thermophilum | | | 68.35 | 85.89 | | 0.81308 |
| Methanoliparia_GoM_asphalt/M.hydrocarbonicum | | | 91.70 | 93.50 | | 0.99131 |
| Methanoliparia_GoM_asphalt/M. thermophilum | | | 68.17 | 85.94 | | 0.82651 |
| M.hydrocarbonicum/Methanoliparia_GoM_oil | | | 92.28 | 93.76 | | 0.99428 |
| M.hydrocarbonicum/Methanoliparia_GoM_asphalt | | | 92.13 | 93.50 | | 0.99131 |
| M.thermophilum/Methanoliparia_GoM_oil | | | 68.36 | 85.89 | | 0.81308 |
| M.thermophilum/Methanoliparia_GoM_asphalt | | | 68.37 | 85.94 | | 0.82651 |
| Syntropho_SAG/S.butanivorans | | | 73.94 | 83.8 | | 0.86 |
| Syntropho_SAG/S.caldarius | | | 75.95 | 84.37 | | 0.94 |
| S.butanivorans/Syntropho_SAG | | | 73.66 | 83.7 | | 0.86 |
| S.caldarius/Syntropho_SAG | | | 76.11 | 84.38 | | 0.94 |

^1^Based on lineage-specific marker genes of *Euryarchaeota* using CheckM.
