# Supplementary material for: Anaerobic Degradation of Non-Methane Alkanes by “Candidatus Methanoliparia” in Hydrocarbon Seeps of the Gulf of Mexico
Source: mBio. 2019 Aug 20;10(4):e01814-19. doi: 10.1128/mBio.01814-19 (PMC6703427; doi:10.1128/mBio.01814-19)
Supplement: TABLE S4 [file mBio.01814-19-st004.docx]

**Table S4.** Representative accession numbers and environmental information from publically available 16S rRNA gene sequences affiliated with *Ca.* Methanoliparia from the SILVA v132 database. Sequence lengths range from 394 to 1451 bp; mbsl= meters below sea level

| **Exemplary NCBI accession number** | **Sampling location** | **Environment** | **Comments** | **Reference** |
| --- | --- | --- | --- | --- |
| AY454634 | Brazil: Santos-Sao Vicente estuary | Brackish sediments with polluted by polycyclic aromatic hydrocarbons | Clone D-C06; alternative name for the clade in previous versions of the SILVA database | (1) |
| AY542189 | Gulf of Mexico, Green Canyon | Marine gas hydrates  (575 mbsl; low temperature) | High percentage of short-chain alkanes | (2) |
| AJ556299 | China: Jurong | Paddy soil |  | (3) |
| DQ521755 | Gulf of Mexico, Green Canyon | Oil-rich mud volcano  (876 mbsl; 6°C) |  | (4) |
| EU735576 | China: Jidong oilfield | Oil-contaminated soils |  | (5) |
| FM866673 | Canada: Cape Breton; Sydney Tar Ponds | Contaminated tar ponds | 281 clones | (6) |
| AM746103 | Gulf of Mexico, Campeche Knolls region | Hydrocarbon-rich marine sediments close to an asphalt volcano  (2902 mbsl) | Named as GoM-Arc2 clade | (7) |
| HQ065902 | Canada: Athabasca oil sand(Alberta) | Oil sand tailing pond |  | (8) |
| HQ845189 | China: Shengli oil field | Methanogenic hexadecane-enrichment culture from oil reservoir sediment | 1600 m below surface | (9) |
| GU120489 | Trinidad y Tobago: Pitch lake, asphalt lake | Liquid asphalts, oil and mud volcanoes | Named as Tar ARC I group; > 50% of all archaea | (10) |
| HM041907 | Japan: Niiboli oilfield | Terrestrial oil reservoir (1050 m below surface) | Present in the crude-oil fraction | (11) |
| AB899898 | Egypt:Suez Gulf, Elzaiteya site | Petroleum-contaminated marine sediment (20 mbsl) |  | (12) |
| JF947128 | China: Shengli oil field | Methanogenic hexadecane-degrading enrichment at 35°C | *Ca.* Methanoliparia represents around 20-40% of the archaeal community. | (13) |
| JF947151 | China: Shengli oil field | Methanogenic hexadecane-degrading enrichment at 55°C | *Ca.* Methanoliparia represents around 10-30% of the archaeal community | (13) |
| KC442804 | China: Xinjian Luliang Oil Field | Water samples from terrestrial oil well |  | (14) |
| EF806_00470 (in RXIF01000002.1) | Brazil | Enrichment culture from petroleum sample (50 °C) | *Ca.* Methanoliparum thermophilum (NM1a) | (15) |
| EF807_00465 (in RXIL01000007.1) | USA, Santa Barbara Channel, oil seep | Oil polluted marine water | *Ca.* Methanolliviera hydrocarbonicum (NM1b) | (15) |
| AY996927 |  | Hydrocarbon-containing wastewater |  | Kapley et al., unpublished* |
| KU025251 |  | Oil field |  | Song, unpublished* |
| LC431890 | Gulf of Mexico, Campeche Knolls region | Solid and liquid asphalt pieces |  | Wegener et al, unpublished* |

*Sequences released in NCBI

**References**

1. Piza FF. 2004. Ecologia molecular microbiana associada a sedimentos do estuário de Santos-São Vicente (SP, Brasil)Universidade Estadual de Campinas (UNICAMP). Instituto de Biologia.

2. Mills HJ, Martinez RJ, Story S, Sobecky PA. 2005. Characterization of Microbial Community Structure in Gulf of Mexico Gas Hydrates: Comparative Analysis of DNA- and RNA-Derived Clone Libraries. Applied and environmental microbiology 71:3235-3247.

3. Wu X-L, Friedrich MW, Conrad R. 2006. Diversity and ubiquity of thermophilic methanogenic archaea in temperate anoxic soils. Environmental microbiology 8:394-404.

4. Lloyd KG, Lapham L, Teske A. 2006. An Anaerobic Methane-Oxidizing Community of ANME-1b Archaea in Hypersaline Gulf of Mexico Sediments. Applied and environmental microbiology 72:7218-7230.

5. Liu R, Zhang Y, Ding R, Li D, Gao Y, Yang M. 2009. Comparison of archaeal and bacterial community structures in heavily oil-contaminated and pristine soils. Journal of Bioscience and Bioengineering 108:400-407.

6. Koenig JE, Sharp C, Dlutek M, Curtis B, Joss M, Boucher Y, Doolittle WF. 2009. Integron Gene Cassettes and Degradation of Compounds Associated with Industrial Waste: The Case of the Sydney Tar Ponds. PLoS One 4:e5276.

7. Orcutt BN, Joye SB, Kleindienst S, Knittel K, Ramette A, Reitz A, Samarkin V, Treude T, Boetius A. 2010. Impact of natural oil and higher hydrocarbons on microbial diversity, distribution, and activity in Gulf of Mexico cold-seep sediments. Deep Sea Research Part II: Topical Studies in Oceanography 57:2008-2021.

8. Ramos-Padrón E, Bordenave S, Lin S, Bhaskar IM, Dong X, Sensen CW, Fournier J, Voordouw G, Gieg LM. 2011. Carbon and Sulfur Cycling by Microbial Communities in a Gypsum-Treated Oil Sands Tailings Pond. Environmental Science & Technology 45:439-446.

9. Cheng L, Dai L, Li X, Zhang H, Lu Y. 2011. Isolation and Characterization of Methanothermobacter crinale sp. nov., a Novel Hydrogenotrophic Methanogen from the Shengli Oil Field. Applied and environmental microbiology 77:5212-5219.

10. Schulze-Makuch D, Haque S, Antonio MRdS, Ali D, Hosein R, Song YC, Yang J, Zaikova E, Beckles DM, Guinan E, Lehto HJ, Hallam SJ. 2011. Microbial Life in a Liquid Asphalt Desert. Astrobiology 11:241-258.

11. Kobayashi H, Endo K, Sakata S, Mayumi D, Kawaguchi H, Ikarashi M, Miyagawa Y, Maeda H, Sato K. 2012. Phylogenetic diversity of microbial communities associated with the crude-oil, large-insoluble-particle and formation-water components of the reservoir fluid from a non-flooded high-temperature petroleum reservoir. Journal of Bioscience and Bioengineering 113:204-210.

12. Elsaied HE. 2014. Genotyping of uncultured archaea in a polluted site of Suez Gulf, Egypt, based on 16S rRNA gene analyses. The Egyptian Journal of Aquatic Research 40:27-33.

13. Cheng L, Shi S, Li Q, Chen J, Zhang H, Lu Y. 2014. Progressive Degradation of Crude Oil n-Alkanes Coupled to Methane Production under Mesophilic and Thermophilic Conditions. PLoS One 9:e113253.

14. Gao P, Tian H, Li G, Sun H, Ma T. 2015. Microbial diversity and abundance in the Xinjiang Luliang long-term water-flooding petroleum reservoir. MicrobiologyOpen 4:332-342.

15. Borrel G, Adam PS, McKay LJ, Chen L-X, Sierra-García IN, Sieber CMK, Letourneur Q, Ghozlane A, Andersen GL, Li W-J, Hallam SJ, Muyzer G, de Oliveira VM, Inskeep WP, Banfield JF, Gribaldo S. 2019. Wide diversity of methane and short-chain alkane metabolisms in uncultured archaea. Nature Microbiology 4:603-613.
