# Supplementary material for: Anaerobic Degradation of Non-Methane Alkanes by “Candidatus Methanoliparia” in Hydrocarbon Seeps of the Gulf of Mexico
Source: mBio. 2019 Aug 20;10(4):e01814-19. doi: 10.1128/mBio.01814-19 (PMC6703427; doi:10.1128/mBio.01814-19)
Supplement: FIG S4 [file mBio.01814-19-sf004.pdf]

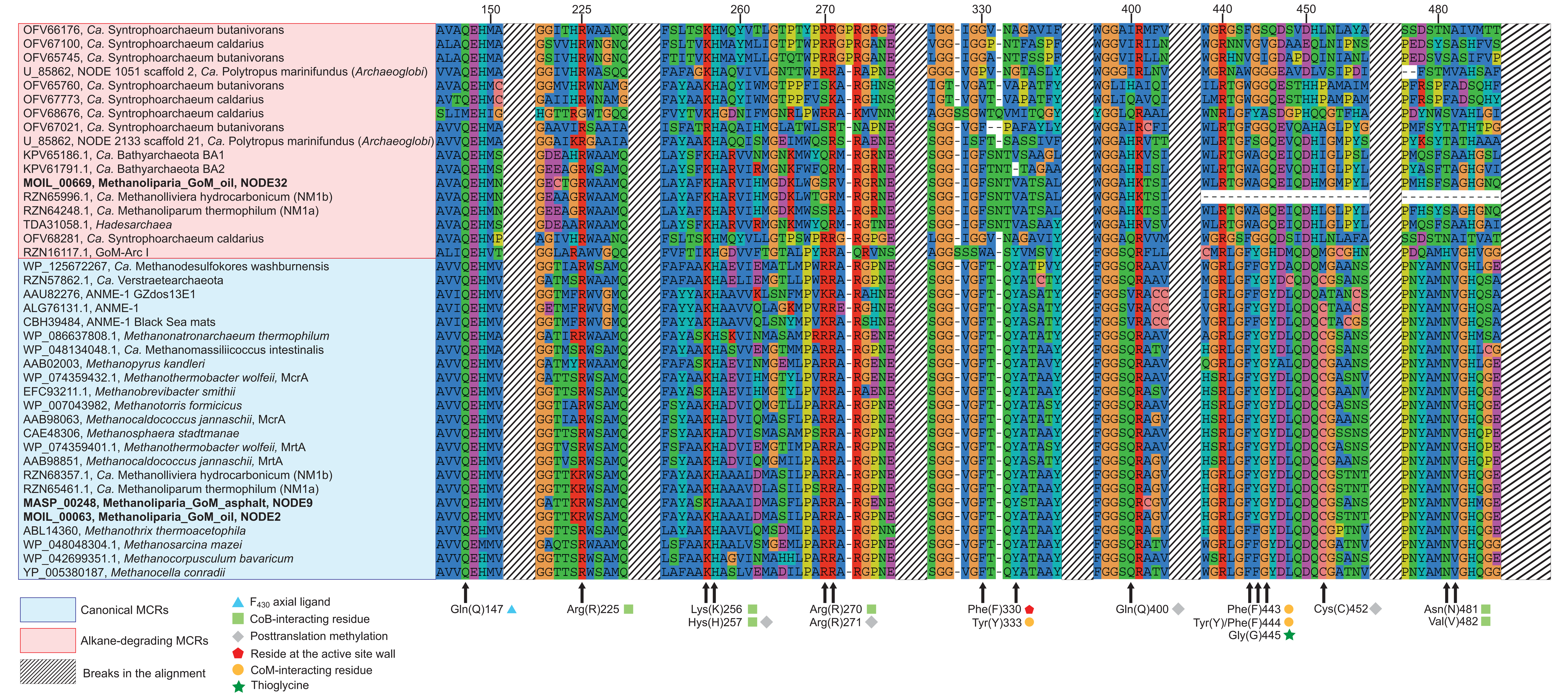
150
225
260
270
330
400
440
450
480
OFV66176, Ca. Syntrophoarchaeum butanivorans
OFV67100, Ca. Syntrophoarchaeum caldarius
OFV65745, Ca. Syntrophoarchaeum butanivorans
U_85862, NODE 1051 scaffold 2, Ca. Polytropus marinifundus (Archaeoglobi)
OFV65760, Ca. Syntrophoarchaeum butanivorans
OFV67773, Ca. Syntrophoarchaeum caldarius
OFV68676, Ca. Syntrophoarchaeum caldarius
OFV67021, Ca. Syntrophoarchaeum butanivorans
U_85862, NODE 2133 scaffold 21, Ca. Polytropus marinifundus (Archaeoglobi)
KPV65186.1, Ca. Bathyarchaeota BA1
KPV61791.1, Ca. Bathyarchaeota BA2
MOIL_00669, Methanoliparia_GoM_oil, NODE32
RZN65996.1, Ca. Methanolliviera hydrocarbonicum (NM1b)
RZN64248.1, Ca. Methanoliparum thermophilum (NM1a)
TDA31058.1, Hadesarchaea
OFV68281, Ca. Syntrophoarchaeum caldarius
RZN16117.1, GoM-Arc I
WP_125672267, Ca. Methanodesulfokores washburnensis
RZN57862.1, Ca. Verstraetearchaeota
AAU82276, ANME-1 GZdos13E1
ALG76131.1, ANME-1
CBH39484, ANME-1 Black Sea mats
WP_086637808.1, Methanonatronarchaeum thermophilum
WP_048134048.1, Ca. Methanomassiliicoccus intestinalis
AAB02003, Methanopyrus kandleri
WP_074359432.1, Methanothermobacter wolfeii, McrA
EFC93211.1, Methanobrevibacter smithii
WP_007043982, Methanotorris formicicus
AAB98063, Methanocaldococcus jannaschii, McrA
CAE48306, Methanosphaera stadtmanae
WP_074359401.1, Methanothermobacter wolfeii, MrtA
AAB98851, Methanocaldococcus jannaschii, MrtA
RZN68357.1, Ca. Methanolliviera hydrocarbonicum (NM1b)
RZN65461.1, Ca. Methanoliparum thermophilum (NM1a)
MASP_00248, Methanoliparia_GoM_asphalt, NODE9
MOIL_00063, Methanoliparia_GoM_oil, NODE2
ABL14360, Methanothrix thermoacetophila
WP_048048304.1, Methanosarcina mazei
WP_042699351.1, Methanocorpusculum bavaricum
YP_005380187, Methanocella conradii
Gln(Q)147
Arg(R)225
Lys(K)256
Hys(H)257
Arg(R)270
Arg(R)271
Phe(F)330
Tyr(Y)333
Gln(Q)400
Phe(F)443
Tyr(Y)/Phe(F)444
Gly(G)445
Cys(C)452
Asn(N)481
Val(V)482
Canonical MCRs
Alkane-degrading MCRs
Breaks in the alignment
F430 axial ligand
CoB-interacting residue
Posttranslation methylation
Reside at the active site wall
CoM-interacting residue
Thioglycine
